# RNA-seq analysis reveals TRPC genes to impact an unexpected number of metabolic and regulatory pathways

**DOI:** 10.1038/s41598-020-61177-x

**Published:** 2020-04-29

**Authors:** Karina Formoso, Sebastian Susperreguy, Marc Freichel, Lutz Birnbaumer

**Affiliations:** 1 Institute for Biomedical Research (BIOMED UCA-CONICET). School of Medical Sciences, Catholic University of Argentina (UCA), Buenos Aires, C1107AFF Argentina; 2 Institute of Physiology and Pathophysiology, Heidelberg University, 69120 Heidelberg, Germany; 3 Neurobiology Laboratory, National Institute of Environmental Health Sciences (NIEHS), Research Triangle Park, North Carolina, 27709 USA

**Keywords:** Cell biology, Computational biology and bioinformatics

## Abstract

The seven-member transient receptor potential canonical genes (TRPC1-7) encode cation channels linked to several human diseases. There is little understanding of the participation of each TRPC in each pathology, considering functional redundancy. Also, most of the inhibitors available are not specific. Thus, we developed mice that lack all of the TRPCs and performed a transcriptome analysis in eight tissues. The aim of this research was to address the impact of the absence of all TRPC channels on gene expression. We obtained a total of 4305 differentially expressed genes (DEGs) in at least one tissue where spleen showed the highest number of DEGs (1371). Just 21 genes were modified in all the tissues. Performing a pathway enrichment analysis, we found that many important signaling pathways were modified in more than one tissue, including PI3K (phosphatidylinositol 3-kinase/protein kinase-B) signaling pathway, cytokine-cytokine receptor interaction, extracellular matrix (ECM)-receptor interaction and circadian rhythms. We describe for the first time the changes at the transcriptome level due to the lack of all TRPC proteins in a mouse model and provide a starting point to understand the function of TRPC channels and their possible roles in pathologies.

## Introduction

The transient receptor potential canonical (TRPC) are non-specific cation channels and the first group to be described among the mammalian TRP family. The TRPC family is composed of seven different proteins (TRPC1-7) encoded in seven different genes, though TRPC2 is a pseudogene in humans^1^. All seven channels share structural features. All of them possess six transmembrane helices, a hydrophobic loop contributing to the outer vestibule and upper gate of the pore, several, three to four ankyrin repeats, coiled-coil domains in the N- and C-termini and, a C-terminal proline-rich region^2,3^. In spite of recent advances in elucidating the atomic structure of TRPCs their physiologic functions are still understood only poorly^4–6^. Based on sequence similarity, the 7 TRPC proteins segregate into 4 subgroups: TRPC1, TRPC4 & 5, TRPC3, 6 & 7 and TRPC2. TRPCs were originally proposed to be activated by store depletion, which may not be so, but, most if not all, TRPCs appear to be activated by diacylglycerol (DAG)^7,8^. In addition to allowing passage of Na+ and K+, TRPC channels allow passage of Calcium. TRPC proteins assemble into cation channels as homo- and hetero-tetramers including 2 or 3 TRPCs subtypes^9–11^. TRPC channels have been linked to various human diseases that show a dysregulation of store-operated Calcium entry (SOCE), such as in cancer^12^, cardiovascular^13^, pulmonary^14^, neuropsychiatric and neurological diseases^15^. Also, TRPC channels have been repeatedly implicated in NFAT activation, particularly in cardiac tissue pathologies^16^. Thus transcriptional regulation by NFAT and/or other transcription factors might be the basis of numerous phenotypes developed in KO mice that resulted from dysregulated expression of genes.

Despite the great increase in the last decades in the number of publications in the TRPC field, the exact function, and mechanism of action of TRPCs have not been fully described. Regarding their role in different pathologies, there is little understanding of the degree to which each TRPC participates in each pathology, considering that they present functional redundancy. Also, it is important to highlight that the inhibitors available for TRPCs are not specific, most of the drugs affect more than one TRPC and in many cases other proteins such as ORAI1, so a thorough study of TRPCs is challenging. As a consequence of the complex scenario found in the field of TRPC research, we developed mice that lack all of the TRPC channels (TRPC hepta knockout mice) that have been experimentally characterized (see refs. ^17,18^). The aim of this work was to address the impact of the absence of all TRPC channels on gene expression in several tissues simultaneously and ask at a global level which functions depend on TRPC channels during their normal functioning.

## Results

In order to determine the relevance of TRPCs expression in different tissues four TRPC heptaKO and four WT three-month-old male mice were used to obtain RNA samples from Cardiac ventricle (Heart), Kidney, Spleen, Testis, Liver lobes (Liver), Brain (Midbrain and Forebrain), and Lung lobes (Lung). From these samples, we obtained 1.95 × 10^9^ 51-nt reads, of which 1.67 × 10^9^ (85%) mapped uniquely to the mouse genome for an average of 26.07 million 51-nt reads per sample, using Illumina Bioanalyzer technology. We found a good identification of the sequence throughout the readings and the quality score per sequence obtained was high (near 40). Overall the data had an appropriate quality to continue the analysis.

**Figure 1:**
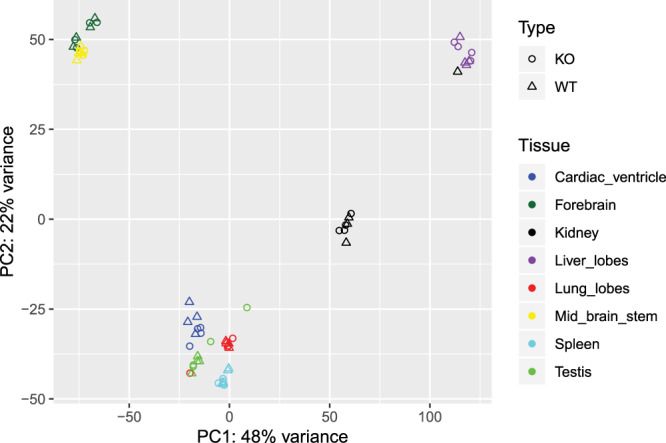
Principal component analysis (PCA) of WT and TRPC heptaKO mouse tissues. PCA plot depicts the distribution of the gene profile of each sample, KO and WT, and each tissue.

**Figure 2:**
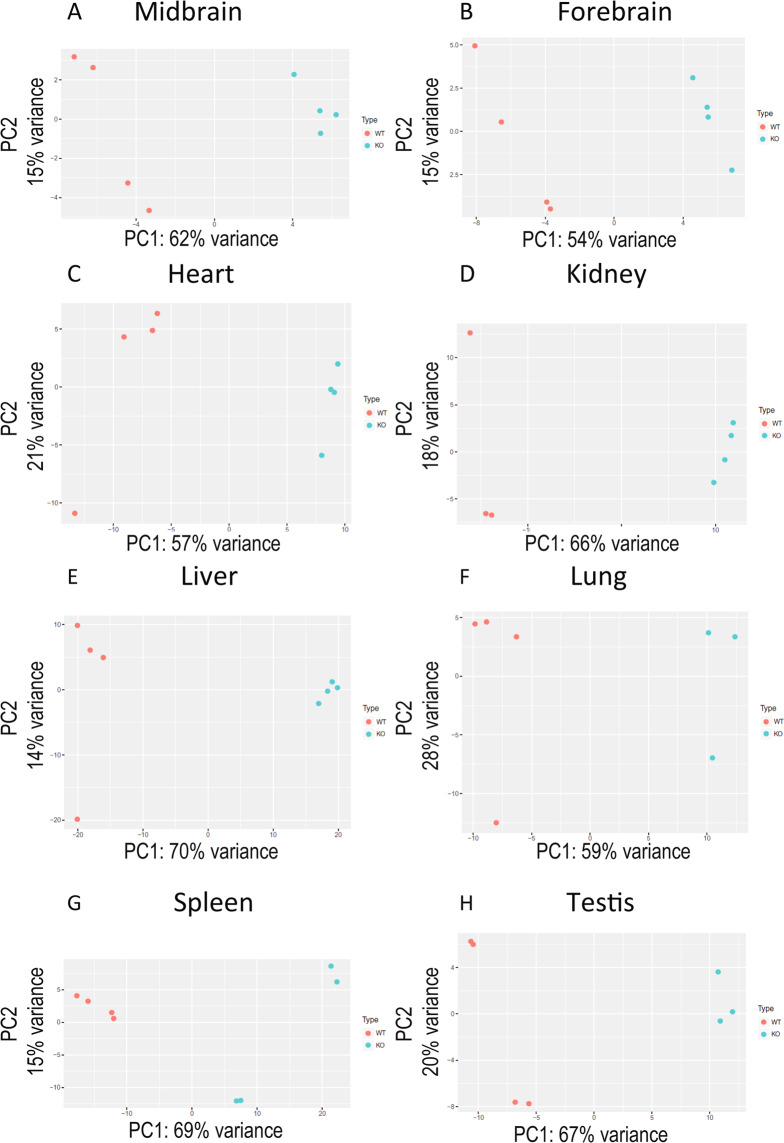
PCA analyses of each sample.

To visualize the variation in expression between samples, we performed a Principal Component Analysis (PCA) where we determined that the principal variation among samples is, as expected, due to the tissue and not to the sample type, eg, WT vs KO (Fig. 1). When the samples were analyzed by tissue, they were segregated by KO or WT phenotype, except for three of the 64 samples (1 WT kidney, 1 KO lung, and 1 KO testis) which were discarded for subsequent analyses (Fig. 2). Our RNAseq analysis allowed us to determine that the number of genes expressed per tissue was between 11.4 and 15.6 thousand.

**Figure 3:**
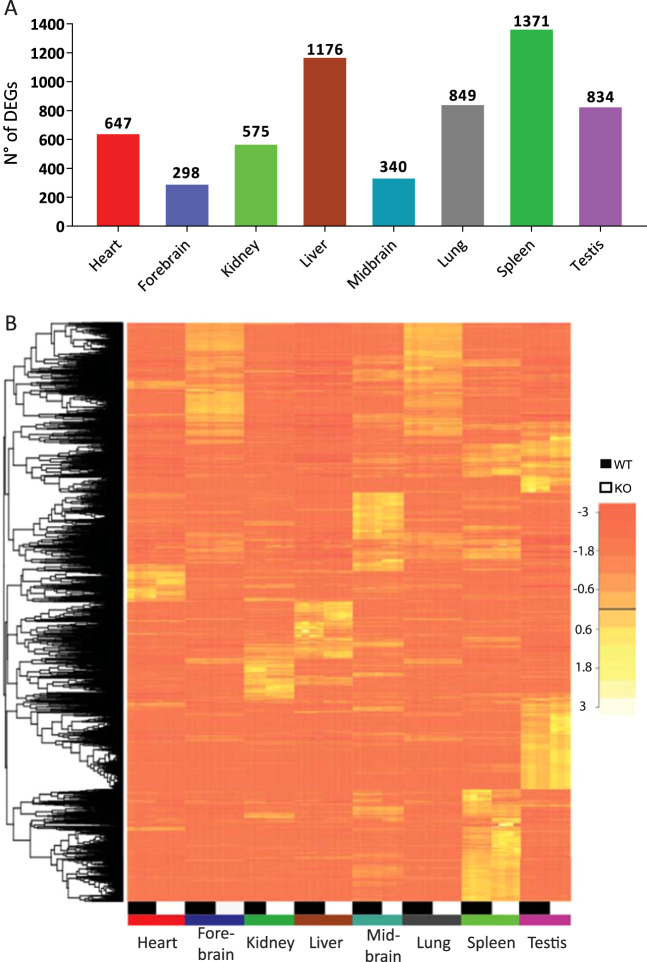
Differentially expressed genes in the KO mice. (**A**) Number of differentially expressed genes (DEGs) per tissue. Bars represent the number of statistically DEGs at p < 0.01 for a 50% change in each tissue. (**B**) Genes with statistically significant alterations in at least one tissue are depicted in a heat map. Each column depicts changes in a WT and a heptaKO and each row a gene.

To determine which genes change their expression between KO and WT, we used edgeR which is an efficient program for determining the most significant Differentially Expressed Genes (DEGs). As a unifying criterion, an adjusted p-value of 0.01 or less and a minimum fold change of 50% was used. The highest differences in the number of DEGs among the tissues were found both in the liver and the spleen (Fig. 3A). A total of 4305 genes were found to be differentially expressed in at least one tissue. From these 77% (3312) are tissue-specific. A heat map that includes these 3312 genes (Fig. 3B), shows both the variability between tissues and also between mice of the WT and KO genotypes. A complete list of differentially expressed genes can be found in Supplementary Table 1.

To analyze the interaction between the identified common targets, a protein-protein interaction (PPI) network was constructed for each tissue using STRING. STRING is a database of known and predicted protein-protein interactions that allows searching in a list of possibly interacting genes or proteins^19^. Next, the network was retrieved and was further analyzed using the Cytoscape software. Cytoscape is an open-source bioinformatics software platform that allows us to visualize and analyze molecular interaction networks. From these analyses, we obtained the top hub proteins, meaning the ones with the highest degree of connectivity, which represents the number of interactions associated with the protein. After that, we used the molecular complex detection plug-in (MCODE) to identify modules, i.e. densely connected nodes. Each module may but need not represent molecular complexes. This is a first approach to determine the possible functions of the proteins affected by the absence of TRPC channels. Finally, we performed a functional enrichment analysis. The aim of this type of analysis was to identify biological issues or functions that represent the differential signals detected. This is carried out through different strategies. The first, over-representation analysis (ORA) seeks to identify which biological pathways are over-represented in the list of DEGs. The second strategy, the network-based gene set enrichment analysis (NGSEA) seeks to identify coordinated signals from genes associated with a given biological subject compatible with interaction networks independently surveyed (eg: metabolic pathway, signaling, gene regulation networks). For each tissue, we report four analyses, one based on Gene Ontology (GO) and three based on the Kyoto Encyclopedia of Genes and Genomes (KEGG) pathways. The ORA analysis was performed both for GO and KEGG. The NGSEA analysis was performed with the Gene Graph Enrichment Analysis (GGEA) and the Signaling Pathway Impact Analysis. (SPIA) both with KEGG pathways. GGEA is a method that allows the search of enrichment in a group of genes, taking into account the inhibitory and excitatory nature between interactions that may exist between them. SPIA analyses whether the expression changes detected are propagated in a manner compatible with the topology of the pathway interactions in question. This analysis allowed us to obtain a list of affected pathways with a false discovery rate (FDR) < 0.05 for each tissue. Table 1 (Metabolic and signaling pathways impacted by the loss of TRPC expression).

**Table 1:** Pathways affected in 2 or more of the tissues analyzed.

	Heart	Brain	Lung	Liver	Spleen	Kidney	Testis
Antigen processing and presentation	X		X			X	
Cell Adhesion			X		X	X	
Circadian regulation of Gene expression	X					X	
Circadian Rhythm	X		X			X	
Cytokine-cytokine receptor interaction		X	X		X		X
ECM-receptor interaction		X	X	X	X	X	X
Focal adhesion		X			X	X	X
Phospholipase D signaling pathway	X						X
PI3K-Akt signaling pathway	X	X	X			X	X
positive regulation of ERK1 and ERK2 cascade			X		X		
PPAR signaling pathway			X	X		X	
Protein digestion and absorption			X		X	X	
Rap1 signaling pathway	X				X		
Ras signaling pathway	X						X

### Analysis of cardiac ventricle reads

**Figure 4:**
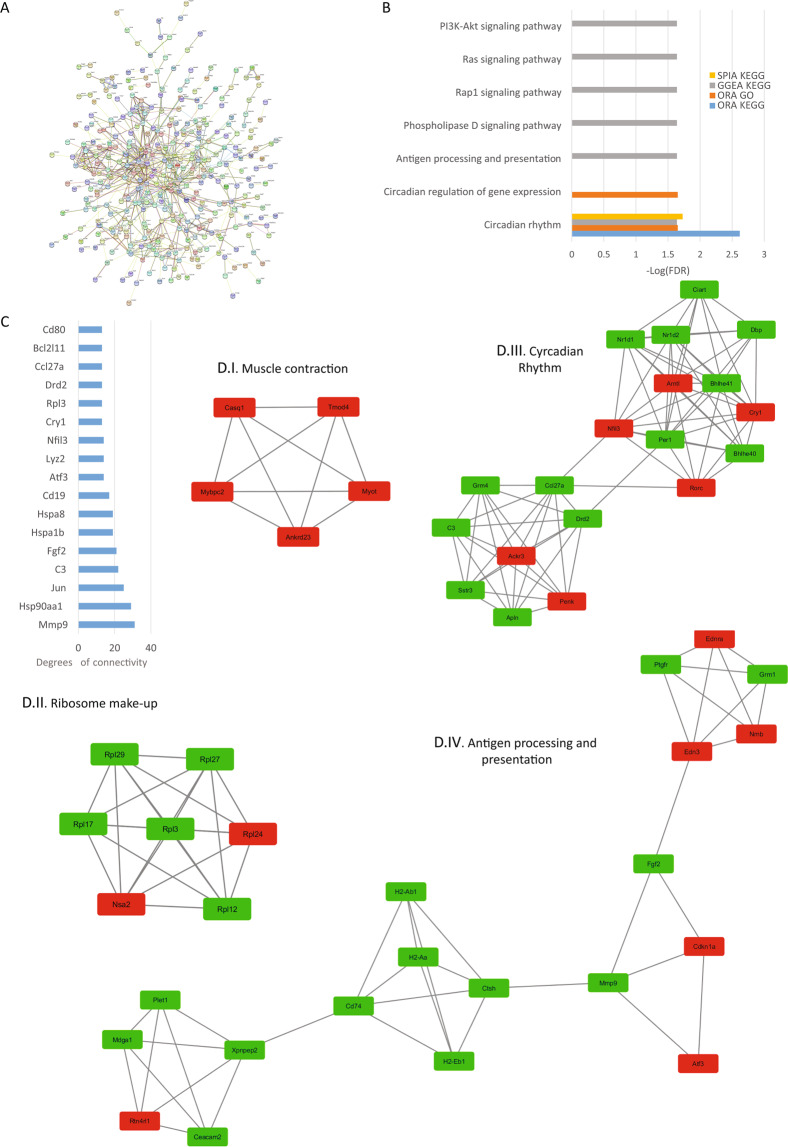
Heart analysis. (**A**) Protein-protein interaction (PPI) network generated with STRING from DEGs in the heart. The network is composed of 482 nodes and connected with 754 edges (p-value of 1.0 e-16). The edges might be drawn in seven different colors indicating different type of evidence (Red line - fusion evidence; Green line - neighborhood evidence; Blue line - cooccurrence evidence; Purple line - experimental evidence; Yellow line - textmining evidence; Light blue line - database evidence; Black line - coexpression evidence). See also high resolution panel A in Supplementary information 2. (**B**) The graph shows the results of the pathway enrichment analyses for the DEGs obtained from the heart. The bars depict the negative logarithm of the false discovery rate (FDR). (**C**) Genes with the highest degree of connectivity obtained from the PPI network with Cytoscape. D.I–IV) Top modules obtained from the PPI network using Cytoscape’s plugin MCODE (Red nodes: Upregulated in hepta KO mice; Green nodes: Downregulated in heptaKO mice).

In the heart, TRPC channel expression is low and is mostly localized to the peripheral plasma membrane in adult cardiac myocytes, though the expression of TRPC channels is increased in cardiac hypertrophy and heart failure. Studies in KO mice showed that they are protected from cardiac hypertrophy and that TRPCs might be contributing to cardiac hypertrophy through the Calcineurin-NFAT pathway^16^. Research from our laboratory established that the absence of TRPC3 and 6 protects mouse hearts in an Ischemia-reperfusion injury model^16^. From the present analysis, we determined that there are 490 protein-coding DEGs with an adjusted p-value < 0.01 and a fold change of at least 50%. The PPI network was retrieved and consisted of 482 nodes and 754 edges with a p-value of 1.0e-16 (Fig. 4A). The top hub proteins were selected from the network with a degree > 13 (Fig. 4C). Among them, we found the matrix metallopeptidase 9 (mmp9). The top 4 significant modules were selected and are shown in Fig. 4D (I–IV). The module I proteins are related to muscle contraction, module II proteins are related to ribosome structure, module III are proteins related to circadian rhythm and finally, module IV are proteins related to antigen processing and presentation. The most affected pathway in the heart was circadian rhythm, in agreement with the PPI network analysis and the role of TRPC6 and 7 seen in double KO mice^20^. Also, Ras, Rap1, PLD signaling and, antigen processing and presentation pathways were significantly affected by the deletion of the TRPC genes (Fig. 4B).

### Analysis of central nervous system reads

**Figure 5:**
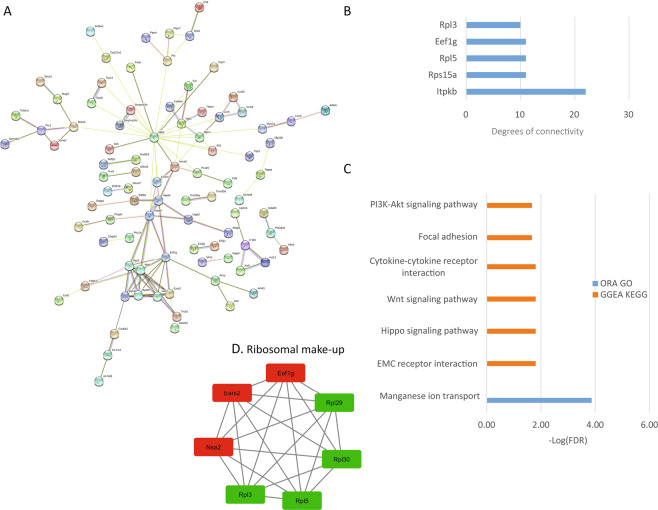
Forebrain analysis. (**A**) PPI network generated with STRING from DEGs in the forebrain. The network comprises 180 nodes and connected with 116 edges (p-value of 0.33). For the rest of the details see Fig. 3 and also high resolution panel A in Supplementary information 2. (**B**) The graph shows the results from the pathway enrichment analyses for the DEGs obtained from the Forebrain. The bars depict the negative logarithm of the false discovery rate (FDR). (**C**) Genes with the highest degree of connectivity obtained from the PPI network with Cytoscape. (**D**) Module obtained from the PPI network using Cytoscape’s plugin MCODE (Red nodes: Upregulated in hepta KO mice; Green nodes: Downregulated in heptaKO mice).

**Figure 6:**
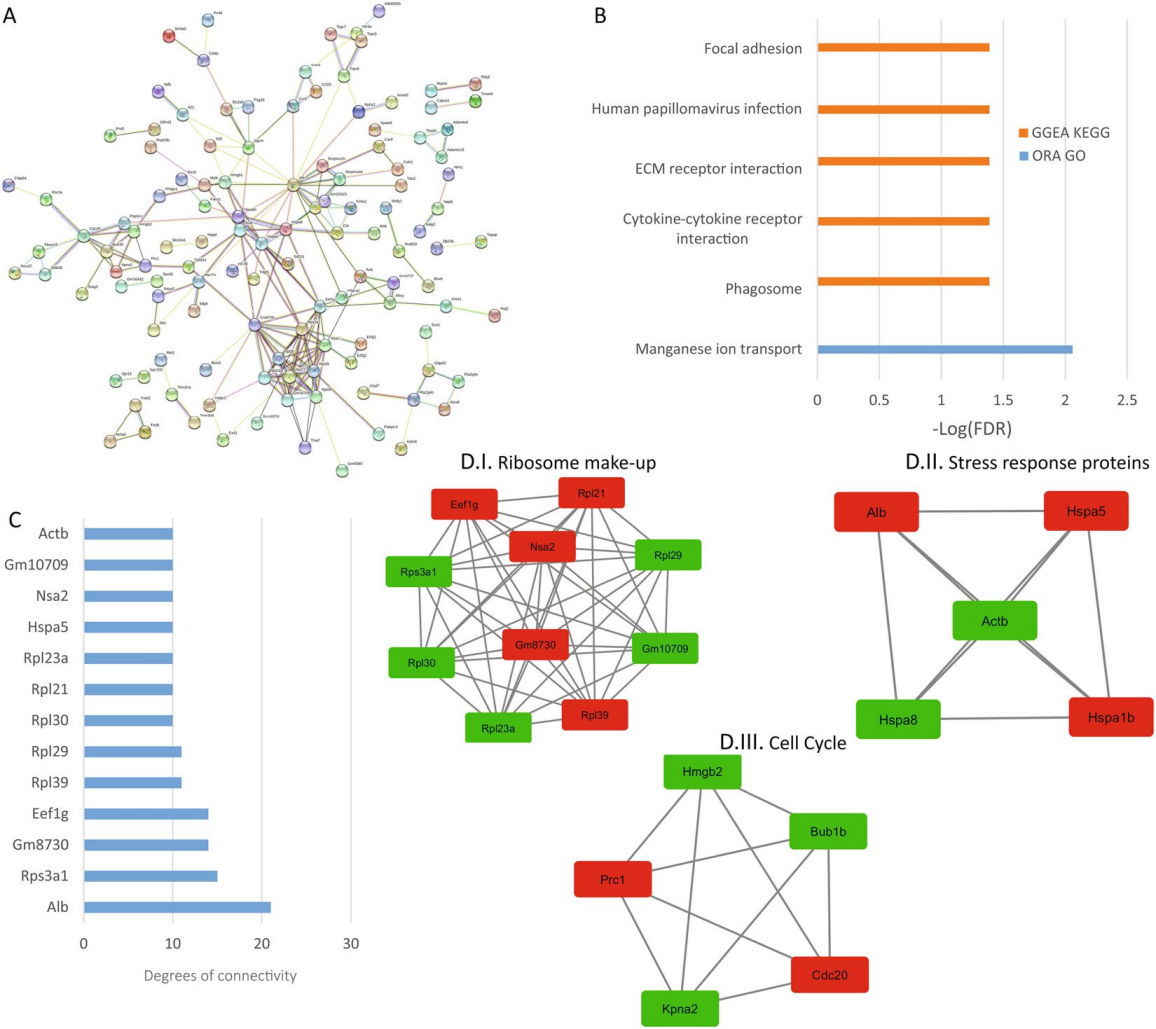
Midbrain (plus brain stem & cerebellum) analysis. (**A**) PPI network generated with STRING from DEGs in the midbrain. The network comprises 216 nodes connected by 209 edges (p-value of 2.77 e-5). For the rest of the details see Fig. 3 and also high resolution panel A in Supplementary information 2. (**B**) The graph shows the results from the pathway enrichment analyses for the DEGs obtained from the Midbrain. The bars depict the negative logarithm of the false discovery rate (FDR). C) Genes with the highest degree of connectivity obtained from the PPI network with Cytoscape. D.I–III) Top three modules obtained from the PPI network using Cytoscape’s plugin MCODE (Red nodes: Upregulated in hepta KO mice; Green nodes: Downregulated in heptaKO mice).

With respect to the nervous system, the expression of TRPCs is ubiquitous and is regulated in different developmental stages^15,21^. Also, TRPCs have been related to several pathologies of the nervous system^15,22^ many related to diverse functions such as neuronal firing, neurite extension and growth cone guidance^21^. We found 185 protein-coding DEGs in the forebrain and 210 DEGs in the midbrain which included basal ganglia and cerebellum. The PPI network retrieved from the forebrain protein-coding DEGs consisted of 180 nodes and 116 edges with a p-value of 0.33, meaning it is not statistically significant, for midbrain, it consisted of 216 nodes, 209 edges and a p-value, of 2.77e-5 (Figs. 5A and 6A, respectively). The top hub proteins were selected from the network (Figs. 5C and 6C). Using Cytoscape and MCODE we obtained 1 module for forebrain and 3 for midbrain (Figs. 5D and 6D) where we found in both a module for ribosomal proteins (5D.I and 6D.I). For midbrain, we found also a module for Stress response proteins (6D.II) and cell cycle proteins (6D.III). From the enrichment analyses, we found that though the modified signaling pathways are few they tend to repeat between forebrain and midbrain, leading to the idea of a conserved functionality of TRPCs throughout the brain (Figs. 5B and 6B). Among them, we found modified focal adhesions, ECM receptor interaction, cytokine-cytokine receptor interaction and, manganese ion transport in both forebrain and midbrain. In forebrain we also found PI3K-Akt and hippo signaling pathway.

### Analysis of kidney reads

**Figure 7:**
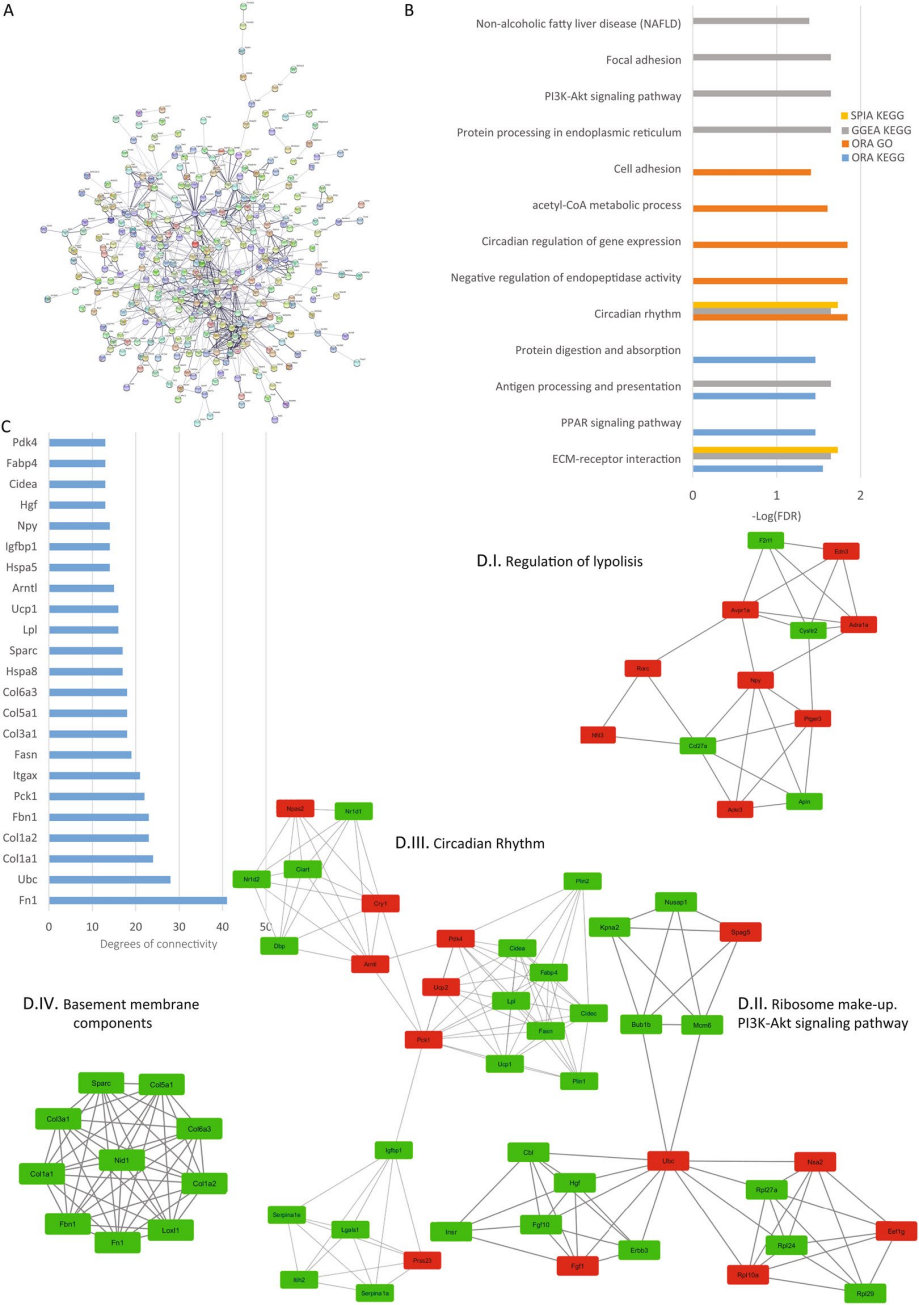
Kidney analysis. (**A**) Protein-protein interaction network generated with STRING from DEGs in the kidney. The network comprises 430 nodes and connected with 812 edges (p-value of 1.0 e-16). For the rest of the details see Fig. 3 and also high resolution panel A in Supplementary information 2. (**B**) The graph shows the results from the pathway enrichment analyses for the DEGs obtained from the Kidney. The bars depict the negative logarithm of the false discovery rate (FDR). (**C**) Genes with the highest degree of connectivity obtained from the PPI network with Cytoscape. D.I–IV) Top four modules obtained from the PPI network using Cytoscape’s plugin MCODE (Red nodes: Upregulated in hepta KO mice; Green nodes: Downregulated in heptaKO mice).

In the kidney, TRPCs 1, 3, 4, 5 and 6 are predominantly expressed in the glomerulus, particularly, in mesangial cells and podocytes^23–28^. Here we found that the kidney showed 433 protein-coding DEGs. The PPI network analyzed with STRING consisted of 430 nodes and 812 edges with a p-value of 1.0e-16 (Fig. 7A). The top hub genes were selected from the network (Fig. 7C). The top 4 significant modules were selected and are shown in Fig. 7D. Module I proteins are related to regulation of lipolysis, Module II is composed mostly of ribosomal proteins and proteins related to PI3K-Akt signaling pathway, Module III has proteins related to circadian rhythm and module IV shows proteins related to basement membrane components. From the pathway enrichment analysis we found ECM-receptor interactions, circadian rhythm, the PI3K signaling pathway similar to the results obtained for heart, and brain, Also, we found focal adhesions, protein digestion and absorption, and antigen processing and presentation. Finding repeated pathways in different tissues gave us the first clue that there are common pathways affected throughout the animal (Fig. 7B).

### Analysis of lung lobe reads

**Figure 8:**
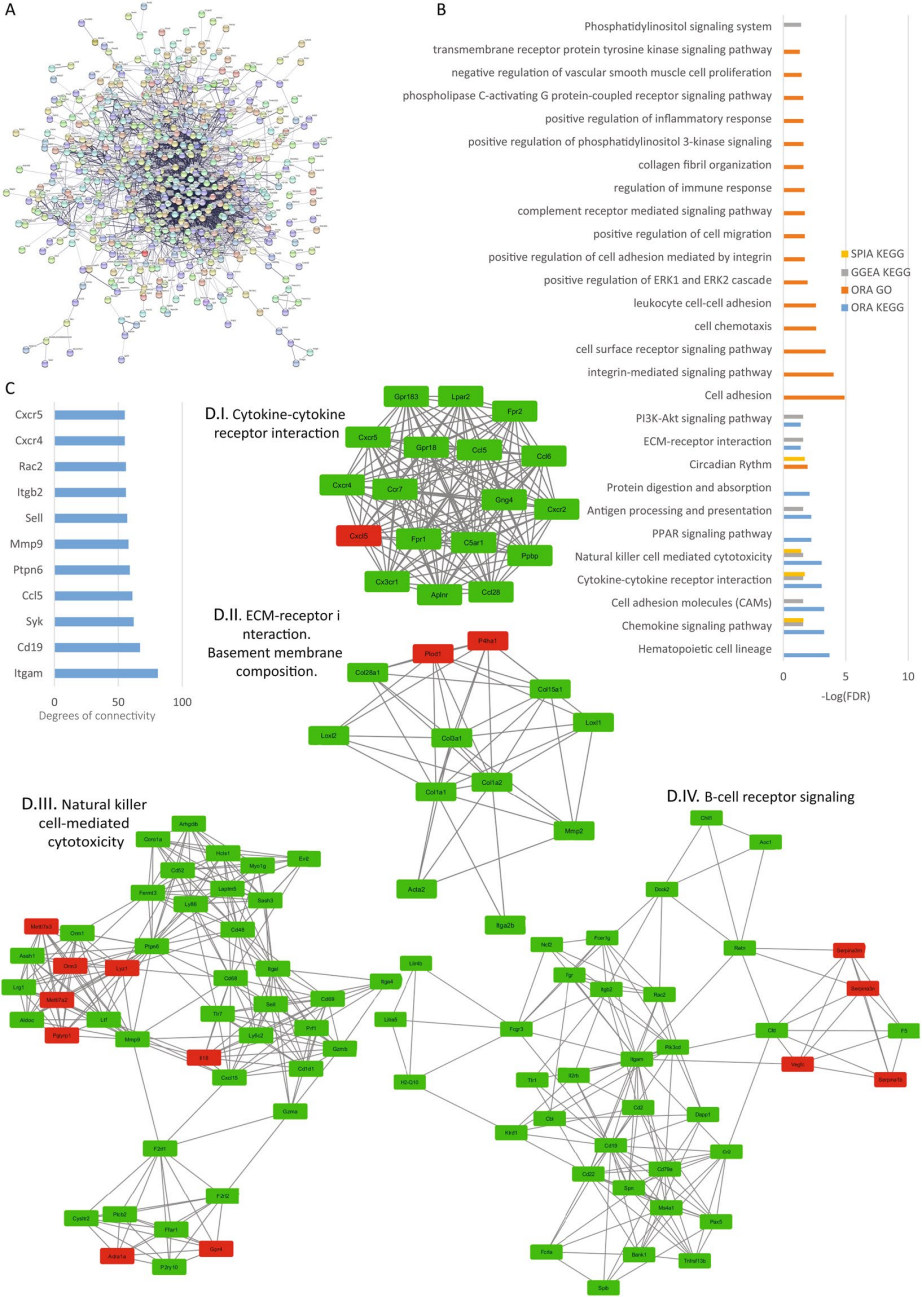
Lung analysis. (**A**) Protein-protein interaction network generated with STRING from DEGs in the lung. The network comprises 658 nodes and connected with 2945 edges (p-value of 1.0 e-16). For the rest of the details see Fig. 3 and also high resolution panel A in Supplementary information 2. (**B**) The graph shows the results from the pathway enrichment analyses for the DEGs obtained from the lung. The bars depict the negative logarithm of the false discovery rate (FDR). (**C**) Genes with the highest degree of connectivity obtained from the PPI network with Cytoscape. D.I–IV) Top four modules obtained from the PPI network using Cytoscape’s plugin MCODE (Red nodes: Upregulated in hepta KO mice; Green nodes: Downregulated in heptaKO mice).

All TRPC isoforms have been detected in the pulmonary vasculature with the highest expression levels for TRPC1 and TRPC6 and lower levels for TRPC3, TRPC 4, TRPC5 and TRPC7^29–32^. Moreover, the expression of TRPC1, TRPC4, and TRPC6 is higher in the proximal pulmonary arteries (which are more sensitive to hypoxia) compared to the distal pulmonary arteries^33^. Lung reads mapped to 676 protein-coding DEGs. The PPI network generated by STRING consisted of 668 nodes and 2945 edges with a p-value of 1.0 e-16 (Fig. 8A). The top hub genes regarding degree were selected from the network (Fig. 8C). Using the Cytoscape software and MCODE plug-in the top 4 significant modules were selected and are shown in Fig. 8D. We found a module related to cytokine-cytokine receptor interaction (8D.I), one related to extracellular matrix and basement membrane composition (8D.II), a third module is related to natural killer cell-mediated cytotoxicity (8D.III) and the last module is related to B-Cell receptor signaling (8D.IV). From the pathway enrichment analyses, we found the ECM-receptor signaling pathway, circadian rhythm, PI3K-Akt signaling pathway, cytokine-cytokine receptor interaction, cell adhesion, and migration, and pathways related to phospholipase D (PLD) signaling to be affected by the loss of TRPCs (Fig. 8B).

### Analysis of testis reads

**Figure 9:**
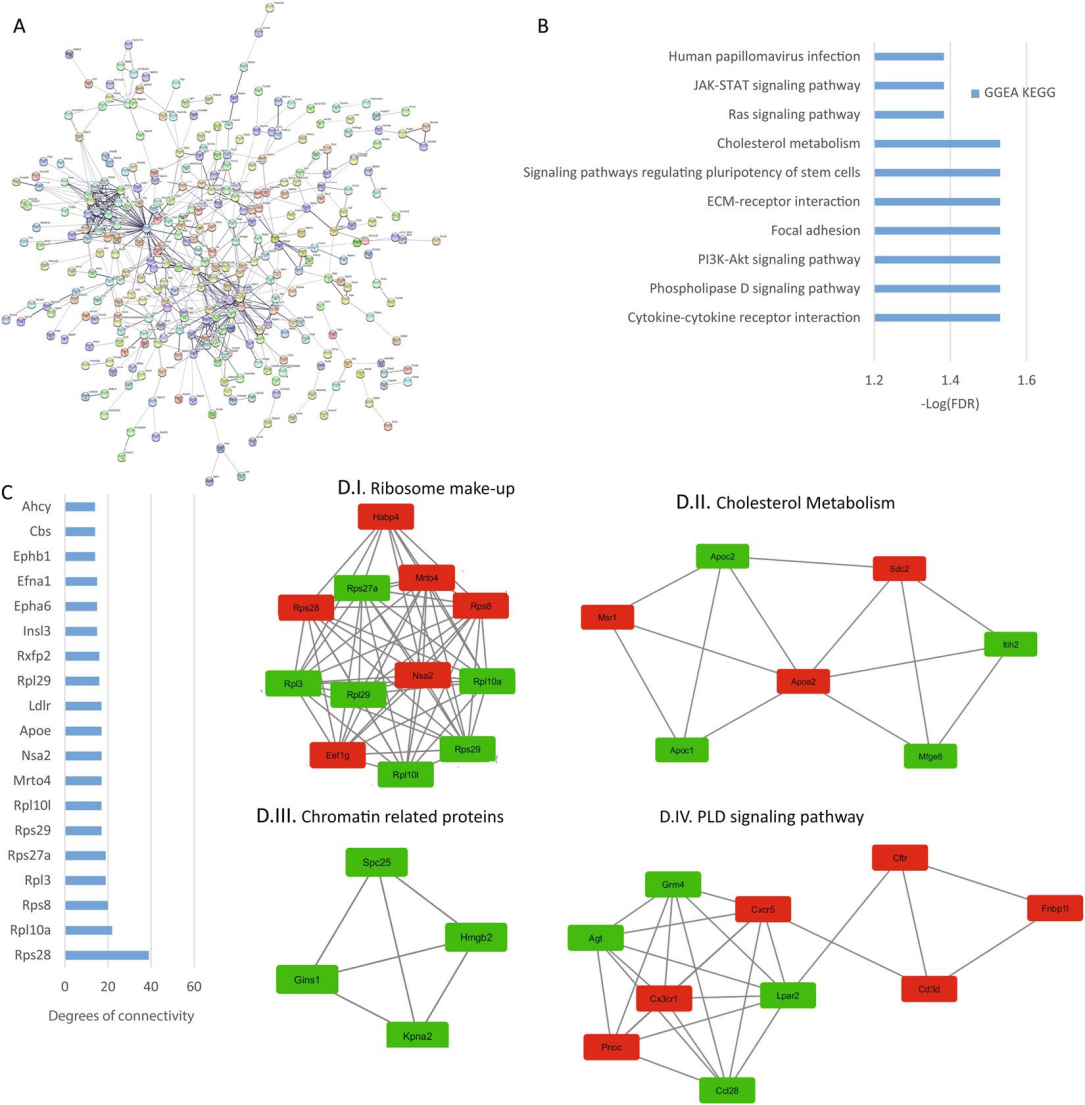
Testis analysis. (**A**) Protein-protein interaction network generated with STRING from DEGs in the testis. The network comprises 483 nodes and connected by 582 edges (p-value of 0.0003). For the rest of the details see Fig. 3 and also high resolution panel A in Supplementary information 2. (**B**) The graph shows the results from the pathway enrichment analyses for the protein-coding DEGs obtained from the testis. The bars depict the negative logarithm of the false discovery rate (FDR). (**C**) Genes with the highest degree of connectivity obtained from the PPI network with Cytoscape. D.I–IV) Top four modules obtained from the PPI network using Cytoscape’s plugin MCODE (Red nodes: Upregulated in hepta KO mice; Green nodes: Downregulated in heptaKO mice).

In the testis, TRPCs reported to be expressed in mammalian spermatozoa include TRPC1, TRPC3, TRPC4, TRPC6, and TRPC2^34^. TRPC1 and TRPC2 have been reported to be present in the acrosomal domain of the mouse sperm head^35,36^. In mouse sperm, TRPC3 has been localized in the post acrosomal head and tail^37^ but in human spermatozoa, TRPC3 is localized on the acrosomal region and mid-piece and, on the plasma membrane of the sperm head and flagella^38^. TRPC4 has not been detected at the protein level in mice but in human spermatozoa, a weak TRPC4 signal was obtained in the head and a strong signal on midpiece and tail^38^. In mouse sperm TRPC6 has been localized in the post-acrosomal head region and sperm flagellum^37^ and in human sperm, TRPC6 has been localized in the mid-piece and the sperm tail^38^. We found 511 protein-coding DEGs that defined a PPI network consisting of 483 nodes and 582 edges with a p-value of 4.03 e -5 (Fig. 9A). The top hub genes regarding degree were selected from the network (Fig. 9C). The top 4 significant modules were selected and are shown in Fig. 9D. Module I has ribosome related proteins, module II is related to cholesterol metabolism, module III is formed by chromatin related proteins, and module IV has proteins related to the PLD signaling pathway. In the pathway enrichment analysis, we only obtained significant differences using GGEA analysis as shown in Fig. 9B which identified ECM-receptor interaction, Focal adhesions, PI3K-Akt, Ras, PLD signaling, Cholesterol metabolism and, cytokine-cytokine receptor interaction to be affected by the loss of TRPCs.

### Analysis of the liver lobe reads

**Figure 10:**
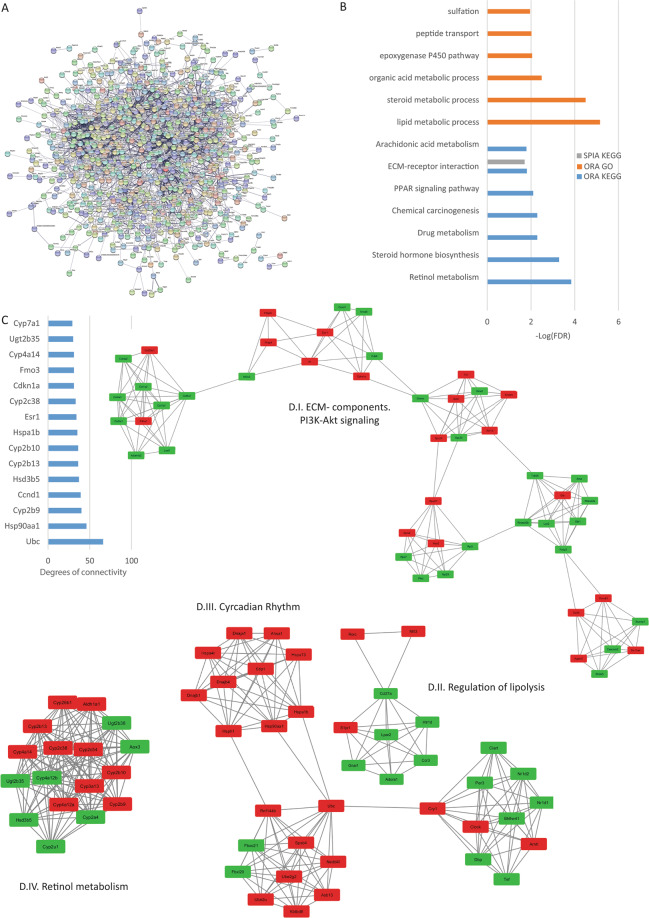
Liver analysis. (**A**) Protein-protein interaction network generated with STRING from DEGs in the Liver. The network comprises 937 nodes and connected with 2691 edges (p-value of 1.0 e-16). For the rest of the details see Fig. 3 and also high resolution panel A in Supplementary information 2. (**B**) The graph shows t the results from the pathway enrichment analyses for the DEGs obtained from the liver. The bars depict the negative logarithm of the false discovery rate (FDR). (**C**) Genes with the highest degree of connectivity obtained from the PPI network with Cytoscape. D.I–IV) Top four modules obtained from the PPI network using Cytoscape’s plugin MCODE (Red nodes: Upregulated in hepta KO mice; Green nodes: Downregulated in heptaKO mice).

In the Liver, TRPC1, TRPC3, TRPC5, and TRPC6 were shown to be expressed^22,39^. From our analysis, 554 protein-coding DEGs were found and the STRING-generated PPI network was formed by 937 nodes and 2691 edges with a p-value of 1.0 e -16 (Fig. 10A). The top hub genes are shown in Fig. 10C and the top 4 significant modules are shown in Fig. 10D where module I is formed by proteins related to ECM components, module II proteins related to regulation of lipolysis, module III is related to circadian rhythm and, finally, module IV is formed by proteins related to retinol metabolism. From the pathway enrichment analysis, we found the PPAR signaling pathway, ECM-receptor interaction, and lipid metabolic processes to be affected by the loss of TRPC expression (Fig. 10B).

### Analysis of spleen reads

**Figure 11:**
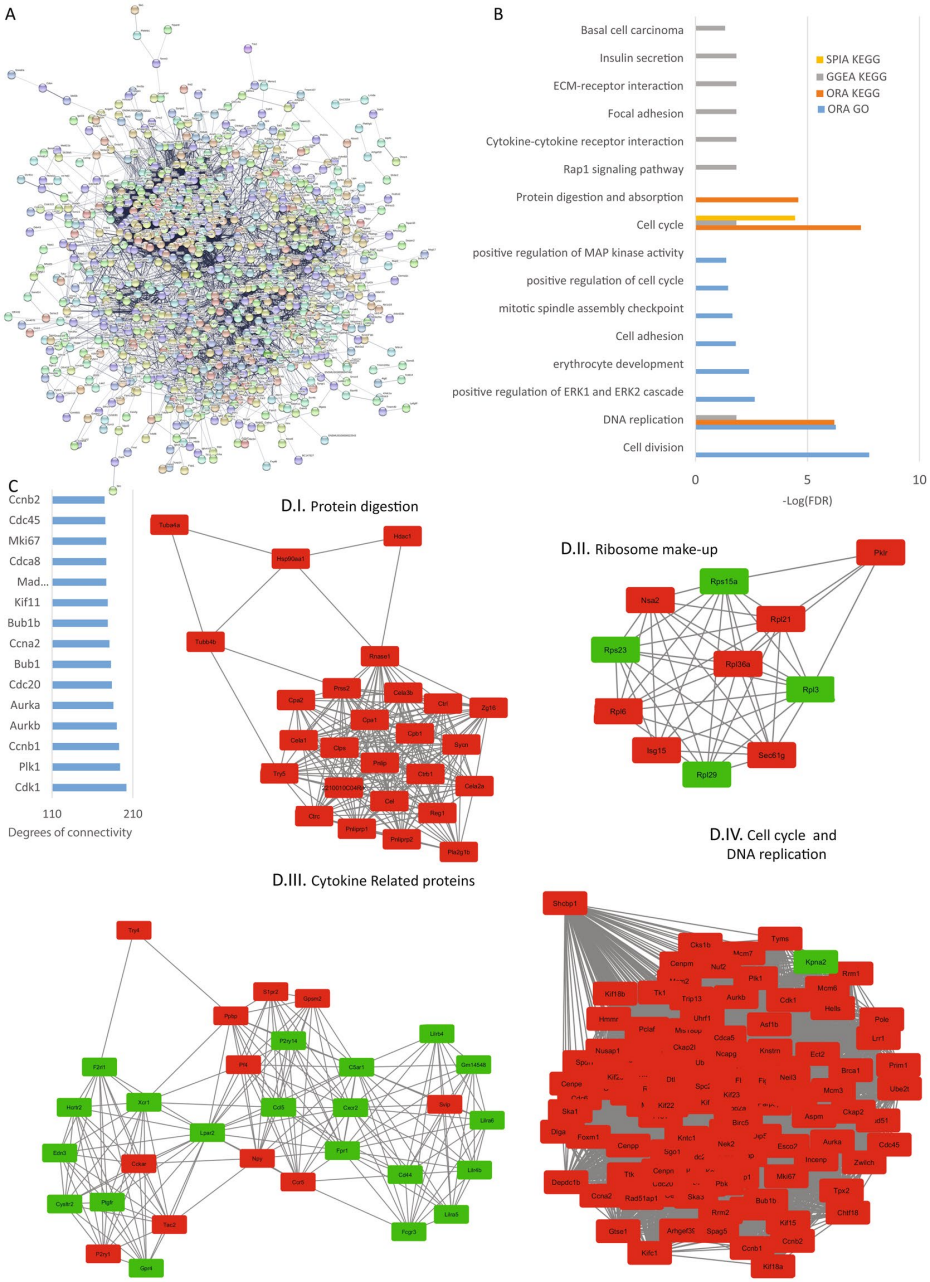
Spleen analysis. (**A**) Protein-protein interaction network generated with STRING from DEGs in the spleen. The network comprises 1015 nodes and connected with 14204 edges (p-value of 1.0 e-16). For the rest of the details see Fig. 3 and also high resolution panel A in Supplementary information 2. (**B**) The graph shows the results from the pathway enrichment analyses for the DEGs obtained from the spleen. The bars depict the negative logarithm of the false discovery rate (FDR). (**C**) Genes with the highest degree of connectivity obtained from the PPI network with Cytoscape. D.I–IV) Top four modules obtained from the PPI network using Cytoscape’s plugin MCODE (Red nodes: Upregulated in hepta KO mice; Green nodes: Downregulated in heptaKO mice).

The bibliography regarding TRPCs in the spleen is limited, but there are publications that propose that all TRPCs are expressed in this tissue^39^. From the 1027 protein-coding DEGs found in this tissue, we obtained a PPI network formed by 1015 nodes and 14204 edges with a p-value of 1.0 e -16 (Fig. 11A). The top hub genes regarding degree are shown (Fig. 11C) and the top 4 significant modules are shown in Fig. 11D. Here we found modules related to protein digestion (11D.I), ribosome components (11D.II), cytokines (11D.III) and, cell cycle and DNA replication (11D.IV). The pathway enrichment analysis identified several affected pathways, among them ECM-receptor interaction, Focal adhesions, cytokine-cytokine receptor interaction and, protein digestion and absorption (Fig. 11B).

**Figure 12:**
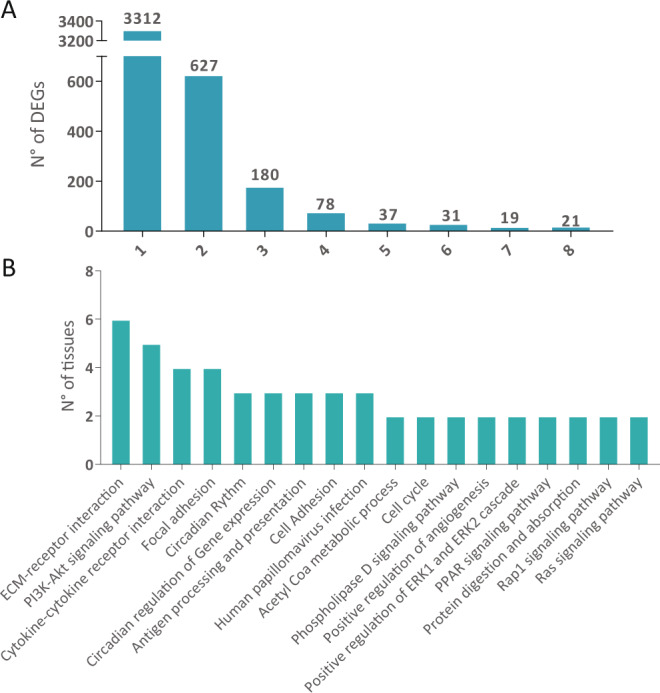
(**A**) Number of differentially expressed genes. Bars depict the number of DEGs found in the indicated number of tissues. (**B**) Enriched pathways most represented in all the tissues analyzed. The bars depict the number of tissues in which the indicated pathways are significantly modified.

We looked for a general modification in all the tissues but the genes modified in 6 and 7 tissues were too few to give a general idea of the processes affected in the whole KO mice. Figure 12A depicts the distribution of DEGs according to the number of tissues involved where we can see that only 21 genes were found to be differentially expressed in all the tissues. From the 40 genes that are differentially expressed in 7 or 8 tissues, we filtered the pseudogenes (Table 2) and analyzed the remaining genes with STRING. Regrettably, we could not find significant interactions among the 21 uniquely differentially expressed genes. Considering this, the pathway enrichment analyses allowed us to search for common pathways affected in different tissues even though the genes modified were not necessarily the same between one and the other. Once each tissue had been analyzed we determined which pathways were represented in the tissues. From this, we determined that the ECM-receptor interaction is affected in most of the tissues. Next is the PI3K signaling pathway, followed by cytokine-cytokine receptor interaction and, focal adhesion (Table 1 and Fig. 12B).

**Table 2:** DEGs Shared by all Tissues.

Gene Symbol	Description
Gdpd3	glycerophosphodiester phosphodiesterase domain containing 3
Trim12a	tripartite motif-containing 12A
mt-Tg	mitochondrially encoded tRNA glycine
Padi4	peptidyl arginine deiminase, type IV
Rpl29	ribosomal protein L29
Trim30d	tripartite motif-containing 30D
Eif3j1	eukaryotic translation initiation factor 3, subunit J1
mt-Nd3	mitochondrially encoded NADH dehydrogenase 3
Kpna2	karyopherin (importin) alpha 2
Jmjd7	jumonji domain containing 7
Capza1	capping protein (actin filament) muscle Z-line, alpha 1
Trim34a	tripartite motif-containing 34A
Zfp457	zinc finger protein 457
Ahcy	S-adenosylhomocysteine hydrolase
Naip5	NLR family, apoptosis inhibitory protein 5

## Discussion

TRPCs have been repeatedly connected to various pathologies^15,22^. For example, TRPC1 has been shown to maintain calcium homeostasis in a mouse model of Parkinson’s disease thus limiting neuronal degeneration. Also, some symptoms are decreased when TRPC1 is expressed in neuronal cells or *in vivo*^40^. A mouse model of Rett syndrome (Mecp2 mutant mice) shows a loss of function of TRPC3 in hippocampus neurons that leads to sensory and motor abnormalities^41^. Increased TRPC6 promoter activity and TRPC6 expression have been linked to the development of idiopathic pulmonary arterial hypertension, which is caused by excessive proliferation of pulmonary artery smooth muscle cells. To date there are several mutations of TRPC6 described to be responsible for the familial forms of focal segmental glomerulosclerosis in man^42–44^. Studies in KO mice showed that they are protected from cardiac hypertrophy and that TRPCs might be contributing to cardiac hypertrophy through Calcineurin-NFAT pathways^45–47^. As mentioned earlier, we reported that the absence of TRPC3 and 6 protects mouse hearts in an Ischemia-reperfusion injury model^16^.

The creation of an animal model devoid active TRPC genes, the TRPC heptaKO mouse, gave us an opportunity to address changes in gene expression in several tissues simultaneously, and to evaluate at a global level which functions depend on TRPC channels during their normal functioning. Based on the 4305 differentially expressed genes in one or more of the tissues examined we performed an enrichment analysis using the KEGG and GO databases, which assemble combinations of expressed genes into diverse well defined cellular processes, and asked which processes were affected in more than one of the tissues of which we analyzed the transcriptome by RNA-seq. We obtained a list of affected pathways that might explain several phenotypes observed in the different KO models of TRPC. The most relevant are discussed next.

One of the pathways we found to be modified is the ECM-receptor interaction. The extracellular matrix is a complex three-dimensional network with multiple components in which cells are located. It is mainly composed of macromolecules such as collagen, elastin, fibronectin, laminins, glycoproteins, proteoglycans and glycosaminoglycans, which are highly acidic and hydrated molecules many of which interact with cell surface receptors. The ECM acts as a physical scaffold interacting with cells through surface receptors, such as integrins, discoidin domain receptors, cell surface proteoglycans, and the hyaluronan receptor CD44. In this way, ECM regulates many cellular processes among them growth, migration, differentiation, survival, homeostasis, and morphogenesis are the most important^48,49^. Changes in the ECM composition are related to various syndromes and pathologies^50^. In this regard, the microenvironment, in which diseases progress, is nowadays considered crucial for the development of pathologic conditions^51,52^. Some specific forms of interaction between ECM and cells are called focal adhesion or focal contacts. These structures, many transmembrane receptors, form the anchor of cellular actin filaments. Other components of focal adhesions are protein kinases and phosphatases that govern signaling pathways that eventually define the cellular processes. Though there is little bibliography supporting the presence of TRPC proteins in cell-ECM junctions, recent work suggests that TRPC7 colocalizes and interacts with syndecan-4 (part of focal adhesion complexes). TRPC7 has been shown to be located in focal adhesions with a similar pattern to that of alpha-actinin-1. Syndecan-4 KO mice show a reduced number of stress fibers and focal adhesions, an effect that can be reversed by removing TRPC7, thus, suggesting that the abnormalities observed were a direct effect of the interaction of Syndecan 4 and TRPC7. Also, consistent with roles in cell adhesion and junction formation, depletion of TRPC4 increased the expression of P-cadherin (cadherin 3) in adherens junctions^53^. It is important to mention that Darier´s disease and psoriatic epidermis that show altered calcium homeostasis also show altered expression of both, P-cadherin and TRPCs^54–57^. Another study found that TRPC5 channels interact through alpha5beta1 integrin with GM1 ganglioside. The integrin then induces the autophosphorylation of its associated focal adhesion kinase (FAK), which in turn activates PLC and PI3K^58^. Some TRPC channels can be activated by the stretching of the membrane^59,60^. TRPC5 and TRPC6 sensing could thus work as osmotic sensor signals to restore hydromineral balance^7,61,62^. TRPC1, for example, is localized in caveolae that are associated with cell shape and actin-controlled changes in membrane tension^63^. The ability to sense changes in the tension of the membrane might thus regulate the formation of focal adhesions.

ECM receptor interactions and focal adhesions are both related to various cellular processes, one of the most important being migration. Migration requires turnover of cell–ECM adhesion complexes with continuous cytoskeleton rearrangement and alterations of cell shape. TRPCs could act in regulating calcium homeostasis and interacting with cell adhesion molecules and other signaling proteins^64^. In this regard, the role of TRPC1 in migration has been described by several groups^65–67^. Upon knockdown of TRPC1, EGF-mediated chemotactic migration is inhibited^68^. TRPC6, on the other hand, promotes cancer cell migration in head and neck squamous cell carcinomas and in glioblastoma^69,70^.

The results obtained here highlight the importance of TRPCs in many functions that depend on the interaction of the ECM with the cells. This allows us to hypothesize a role of TRPCs in cancer progression and other pathologies.

Several studies relate TRPCs to PI3K-Akt signaling pathways. The PI3K/Akt signaling pathway is a major intracellular pathway that among other cellular processes regulates survival, cell growth, differentiation, cellular metabolism, and cytoskeletal reorganization of cells in response to different signals. Akt is downstream of PI3K and is activated through phosphorylation of two residues (serine 473 and threonine 308). PI3Ks are activated by growth factors through GPCR or RTK receptors. The PI3K activation results in the conversion of PtdIns(4,5)P2 (PIP2) to PtdIns(3,4,5)P3 (PIP3), a process that is reversed by the phosphatase PTEN. Then PIP3 interacts with the PH domain of Akt causing its retention at the plasma membrane where it is phosphorylated by 3-phosphoinositide-dependent protein kinase 1 (PDK1) at Thr308 and by PDK2 at Ser 473 resulting in activation of Akt’s kinase activity. Akt subsequently phosphorylates a variety of proteins that lead to different cellular responses^71,72^. This pathway is also dysregulated in various pathologies such as different types of cancer and augmented drug resistance^73^. Activation of TRPC6 in bronchial epithelial cells by LPS/TLR4 and in renal glomerular mesangial cells through cAMP involves PI3K/Akt^74,75^. Evidence also shows that PI3K-mediated signaling pathways are essential in endothelin A (ETA) receptor-evoked TRPC1/C5/C6 and TRPC3/C7 channel activation^76^. In agreement with this, soluble Klotho (klotho) protein present in the systemic circulation inhibits TRPC6 currents in cardiomyocytes by blocking phosphoinositide-3-kinase-dependent exocytosis of TRPC6 channels^77^. Moreover, TRPC1, TRPC5, and TRPC6 translocation to the plasma membrane was found to be dependent on PI3K pathways in different types of cells^61,78–80^. These studies suggest an upstream role for TRPCs activating the PI3K/Akt pathway. In agreement, activating TRPC6 with hyperforin leads to the activation of Ras/MEK/ERK, PI3K/Akt, and CAMKIV kinases and these cues are necessary to promote TRPC6 mediated synaptic plasticity in PC12 cells and primary hippocampal neurons^81^. Also, VEGF can activate TRPC3 and this is followed by PI3K/Akt activation^82^. The administration to mice of M084, an inhibitor of TRPC4/5 increased the phosphorylation levels of Akt and ERK in the prefrontal cortex^83^. Consistent with these findings from our laboratory showed that in cardiomyocyte cultures from triple KO mice (TRPC3/6/7 −/−) pAkt was reduced compared to WT^16^. Also, TRPC1 was found to regulate positively pAkt in PTEN deficient breast cancer cell lines and the lack of TRPC1 or the inhibition of calcium entry reduces Akt phosphorylation and delays muscle cell differentiation^84,85^. Though the evidence does not allow for resolution as to whether TRPC6 activates PI3K/Akt or it is the other way around, it is clear that there is a direct link between the PI3K/Akt pathway and the TRPC protein family.

To our knowledge, there is no experimental data that relate to TRPC channels with circadian rhythm in mammals to date. For this reason, the results presented here are novel and present a new possible function for these proteins not previously reported. The c*ircadian rhythms* are periodic oscillations that occur within 24 hours and determine the sleeping and awakening times, affecting, thus, the whole organism. They are commonly known as an internal biological clock that has components that are common among species and others that vary^86^. These internal clocks work in mammals as negative and positive transcriptional–translational feedback loops where the first negative feedback loop is a rhythmic transcription of period genes (PER1, PER2, and PER3) and cryptochrome genes (CRY1 and CRY2). PER and CRY proteins form a heterodimer, which acts on the CLOCK/BMAL1 heterodimer to repress its own transcription^87^. Phosphorylation of PER and CRY proteins by casein kinase epsilon (CKIepsilon), produces their degradation and restarts the cycle. Then a positive feedback loop initiates the transcription of genes that contain E-box cis-regulatory enhancer sequences and involves CLOCK/BMAL1 heterodimer^87,88^. Reinforcing our findings, it is worth noting that a function for TRPC homologs in Drosophila circadian rhythm has been previously reported^89^. On the other hand, the oscillator circuits do not include a precise timer and constant darkness or constant light leads animals to lose their circadian rhythms. Entrainment and maintenance of circadian rhythms depend on two TRPCs, TRPC6, and TRPC7 which are activated in intrinsically photosensitive Retinal Ganglion Cells (ipRGCs) that project to the suprachiasmatic nucleus (SCN). In ipRGCs light-sensitive melanopsin (Opsin4), a Gq protein-coupled receptor activates phospholipase Cbeta4, which in turn leads to activation of the heteromeric TRPC6/7 channel and cell depolarization^20^. The resulting action potential drives the activity of neurons of the SCN in which the levels of the PER, CRY, CLOCK, and BMAL1 proteins oscillate. In mammals, circadian rhythm controls several processes including heartbeat, sleep-wake cycles, and functions of the immune system and metabolism^90^. Our results show important alterations in the expression of the clock genes but if these changes would result in an altered circadian rhythm needs be studied in the animal model for further corroboration and to determine and describe the phenotype(s) associated with such a dysregulation.

Modifications in intracellular calcium concentrations represent a fundamental mechanism in the control of inflammatory and immune cell functions and the study of the role of TRPC in the innate and adaptive immune response has grown in the last years. One of the pathways modified in the TRPC KO mice is Cytokine-Cytokine receptor interaction. Recent evidence has shown a role for TRPC in several cellular mechanisms of potential significance for the pathophysiology of the innate immune response. TRPC1 in the endothelium increases vascular permeability after TNF/thrombin stimulation^91^. Studies in animal models show that TRPC1 may control IL1β release from macrophages^92^. Similarly, TRPC1 appears to affect the late effects of anaphylaxis by controlling TNF release from mast cells^93^. Another study suggests that mechanical stretch may induce an influx of Calcium and up-regulation of IL-13 and MMP-9 expression in 16HBE cells via activation of TRPC1^94^. TRPC1 is also involved in the inflammatory response to bacterial infection through the TLR4/TRPC1/NF-kB signaling route^95^. In addition, TRPC1 appears to contribute to the regulation of the epithelial-mesenchymal transition (EMT) in cancer; its inhibition suppresses TGF-β1-induced EMT^96,97^. TRPC3 is expressed in various immune cells and has been proposed to contribute to vascular inflammation^98–100^. Also, the brain-derived neurotrophic factor (BNDF) has the effect of upregulating TRPC3 in the cell membrane. This effect has been proposed to avoid neuronal inflammation and myocardial injury^101–103^. Monocytes from patients with hypertension showed an increased TRPC3 expression that was significantly associated with increased expression IL-1beta or TNF-alpha^104^. Alawi *et al*. found that TRPC5 in inflammatory joint conditions was related to an endogenous anti-inflammatory/analgesic pathway. The same study determined that in human inflammatory arthritis chronic treatment with a TRPC5 antagonist reduced TRPC5 mRNA at the same time that augmented the asymmetry, and elevated cytokine concentrations^105^. TRPC5 channels are do not express in resting CD4+ and CD8+ T-cells but are hyper expressed activated cells where they increase Calcium influx^106^. TRPC6 is the most widely studied TRPC in relation to the immune system. TRPC6 is expressed in various cell types (neutrophils, lymphocytes, platelets, and endothelium)^107^. TRPC6 has a role in neutrophil mobilization and promotes the loss of endothelial junctions during acute inflammation^108–111^. In animal models histamine-induced vascular leakage is dependent on TRPC6^112^. TRPC6 cooperates in lipopolysaccharide-induced endothelial activation after being itself activated by increasing intracellular concentrations of diacylglycerol (DAG) downstream of the activation of Toll-like receptor 4 (TLR4^113^). Using a human intestinal myofibroblast model and biopsy samples from patients with cardiovascular disease (CD)), it was shown that although increased TRPC6 (and presumably TRPC4) activity promotes the TGFbeta1-mediated expression of alpha-Smooth Muscle Actin (a-SMA) and N-cadherin and strengthens interactions between the 2 molecules, it also negatively regulates collagen synthesis and the secretion of antifibrotic factors, such as IL-10 and IL-11. TGF-beta1-increased Calcium influx through TRPC6 channels, regulating negatively TGF-beta1-Smad/p38-MAPK/ERK1/2 signaling through calcineurin activation^114^. It was also described that TRPC6 channels in Human Hepatocellular Carcinoma Cells are involved in migration and invasion in response to TGF-β^115^. Cytokine complexes are widely studied as target molecules for different pathologies. The difficulty in their use will be that the many functions cytokines have can lead to undesirable secondary effects.

This is why targeting TRPC proteins might be of interest to modulate immune responses and inflammations driven by cytokine-cytokine receptor interaction.

## Conclusion

There is a vast bibliography relating TRPC channels to different pathologies many of which were obtained from *in vivo* analysis of KO mice. For example, TRPC1 KO mice showed reduced secretion of salivary fluid^116^, a modest decrease in osteoclastogenesis and an increase in bone mass^117^, and movement deficits related to widespread neuronal loss including dopaminergic neurons in basal ganglia^118^. TRPC2 is a pseudogene in humans but in mice is highly expressed in the sensory terminals of the vomeronasal organ. Coherently, the lack of TRPC2 leads to behavioral changes such as loss of sexual discrimination, and impaired maternal aggression (offspring protection)^119,120^. TRPC3 knockout mice show impaired motor coordination^121^ and TRPC6 knockout mice display reduced litter sizes and structural changes of the placenta^122^. All these results led us to hypothesize that mice lacking all seven TRPCs would probably die at early embryonic age or at least have severe developmental defects. To our surprise TRPC heptaKO mice are alive and breeding, through extensive analyses have not been done as yet. Nonetheless, through the present RNA-seq analysis we detected several pathways that are dysregulated compared to the WT mice which however do not lead to the death of the mice. Having said this, we need to keep in mind that many pathologies are related to failure in the transport of calcium through the PM and the ER and many involve increases in intracellular calcium.

We found several pathways dysregulated both in general and in a tissue-specific way. Our work provides the first description of TRPC KO mice at the RNA level providing a starting point to understand the function of TRPC channels and their possible roles in pathologies where their expression is affected.

## Materials and Methods

### Experimental animals

Wild type mouse strains were either 129SvEv, C57Bl/6J or crossbreeds between 129SvEv and C57Bl/6J. TRPC2−/− mice and TRPC4−/− mice were backcrossed for more than 5 generations to C57Bl6J or C57Bl/6N, respectively and then crossed into 129SvEv xC57Bl/6J mice carrying the disrupted alleles of combinations of the TRPC1, TRPC3, TRPC5, TRPC6, and TRPC7 genes. The creation of the individual disrupted TRPC alleles and their validation has been previously described (TRPC1^32^; TRPC2^120^; TRPC3^121^; TRPC4^123^; TRPC5^124^; TRPC6^125^; TRPC7^126^).

Mice were housed in the Association for Assessment and Accreditation of Laboratory Animal Care (AAALAC)-accredited, specific pathogen-free vivarium of the Comparative Medicine Branch of the NIEHS. Animals were treated in compliance with the Guide for the Care and Use of Laboratory Animals (National Academy of Sciences). All procedures and crosses to which the mice were subjected were approved by the NIEHS’s Animal Care and Use Committee (ACUC).

### Isolation of RNA from tissues

Four TRPC KO and four WT three-month-old male mice were used to obtain RNA samples from 7 tissues: Heart, Kidney, Spleen, Testis, Liver, Lung and Brain. The brain was split into two fractions: Forebrain and the remainder of the CNS including brainstem and cerebellum collectively referred to as MidBrain for a total of 64 samples. In several cases, the Forebrain and Midbrain data were pooled and are presented as Brain Mice were euthanized in CO2 chambers and their tissues rapidly dissected, rinsed in ice-cold Dulbecco’s PBS, placed into ice-cold 15-mL Corex tubes with aliquoted RLT solution supplied in RNAeasy Kits (QIAGEN cat #74106) and homogenized with the aid of a Polytron homogenizer fitted with a PT-10 generator. Total RNA was isolated following RNAeasy Kit instructions which include on-column DNAse treatment and sent to Expression Analysis (Research Triangle Park, North Carolina (www.ExpressionAnalysis.com)) for sequencing by the paired-end method. RNA quality was assessed by electrophoresis and bioanalyzer prior to RNA sequencing. An input of 100 ng of total RNA was used to construct cDNA libraries (TruSeq Stranded mRNA Sample Prep Kit, Illumina, #RS-122-2103) following the manufacturer’s instructions. The data analyzed during the current study was deposited in the National Centre for BiotechnologyInformation-Sequence Read Archive (NCBI-SRA) repository with the accesion number PRJNA606559 (www.ncbi.nlm.nih.gov/sra/PRJNA606559).

Twenty-five million 51 bp reads were obtained by the RNA-seq paired-end method with Illumina Bioanalyzer technology. After sequencing, raw data were obtained in the fastq format. FastQC was used for validating the quality of the data. To correct read errors/bias Samtools and trimgalore were used. The data was then aligned to genome ensembl38 using STAR. In order to visualize the variation in expression between samples, we performed a Principal Component Analysis (PCA) using the programming environment R.

### Differential gene expression

The quantification of number and density of reads at the gene level, as well as the differential expression analysis, were carried out under contract by Ariel Chernomoretz, Fundación Instituto Leloir, Buenos Aires,Argentina, with the software ASpli (https://bioconductor.org/packages/release/bioc/html/ASpli.html) and the edgeR package of the statistical programming environment R. The DEGs were identified requiring a fold change greater than 50% and an adjusted p-value (Hochberg-Benjamini multiple test correction) to achieve a false discovery rate < 0.01 (adjusted p-value < 0.01)^127^.

### Protein-protein interaction and pathway enrichment analysis

The protein-protein interaction (PPI) networks were constructed using STRING (https://string-db.org/) for each tissue^19^. Next, we used the Cytoscape software^128^ to obtain the top hub proteins were selected from the network. The molecular complex detection plug-in (MCODE) was used to obtain the modules. The criteria used were Degree cut-off = 2, node score cut-off = 0.2, k-core = 2, and max Depth = 100. The top modules were selected for graphical representation.

The pathway enrichment analysis was performed with two strategies implemented in the EnrichmentBrowser and cluster profile packages: the over-representation analysis (ORA) and the network-based gene set enrichment analysis (NGSEA). The ORA analysis was performed both for GO and KEGG. On the other hand, the NGSEA analysis was performed with the Gene Graph Enrichment Analysis (GGEA) and the Signaling Pathway Impact Analysis (SPIA) using KEGG pathways for both cases. The pathways considered significantly changed had a false discovery rate (FDR) < 0.05.

## Supplementary information

Supplementary Information.

Supplementary Information 2.
