# Supplementary material for: RNA-seq analysis reveals TRPC genes to impact an unexpected number of metabolic and regulatory pathways
Source: Sci Rep. 2020 Apr 29;10:7227. doi: 10.1038/s41598-020-61177-x (PMC7190874; doi:10.1038/s41598-020-61177-x)
Supplement: Supplementary file 2 — Supplementary Information 2. [file 41598_2020_61177_MOESM2_ESM.pdf]

RNA-seq analysis reveals TRPC genes to impact an unexpected number of metabolic and regulatory pathways

Karina Formoso [1,4], Sebastian Susperreguy[1], Marc Freichel [2] & Lutz Birnbaumer[1,3*]

[1]Institute for Biomedical Research (BIOMED UCA-CONICET). School of Medical Sciences, Catholic University of Argentina (UCA), Buenos Aires, C1107AFF, Argentina.

[2]Institute of Physiology and Pathophysiology, Heidelberg University, 69120, Heidelberg, Germany.

[3]Neurobiology Laboratory, National Institute of Environmental Health Sciences (NIEHS), Research Triangle Park, North Carolina, 27709, USA.

[4] Karina Formoso is a postdoctoral research fellow of the National Scientifc and Technical Research Council (CONICET).

*email: birnbau1@gmail.com

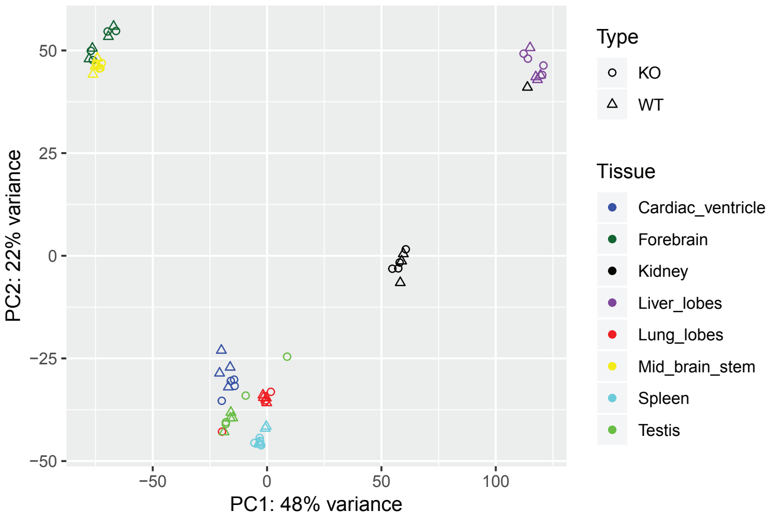

PC2: 22% variance
PC1: 48% variance
50
25
0
−25
−50
−50
0
50
100
Type
KO
WT
Tissue
Cardiac_ventricle
Forebrain
Kidney
Liver_lobes
Lung_lobes
Mid_brain_stem
Spleen
Testis

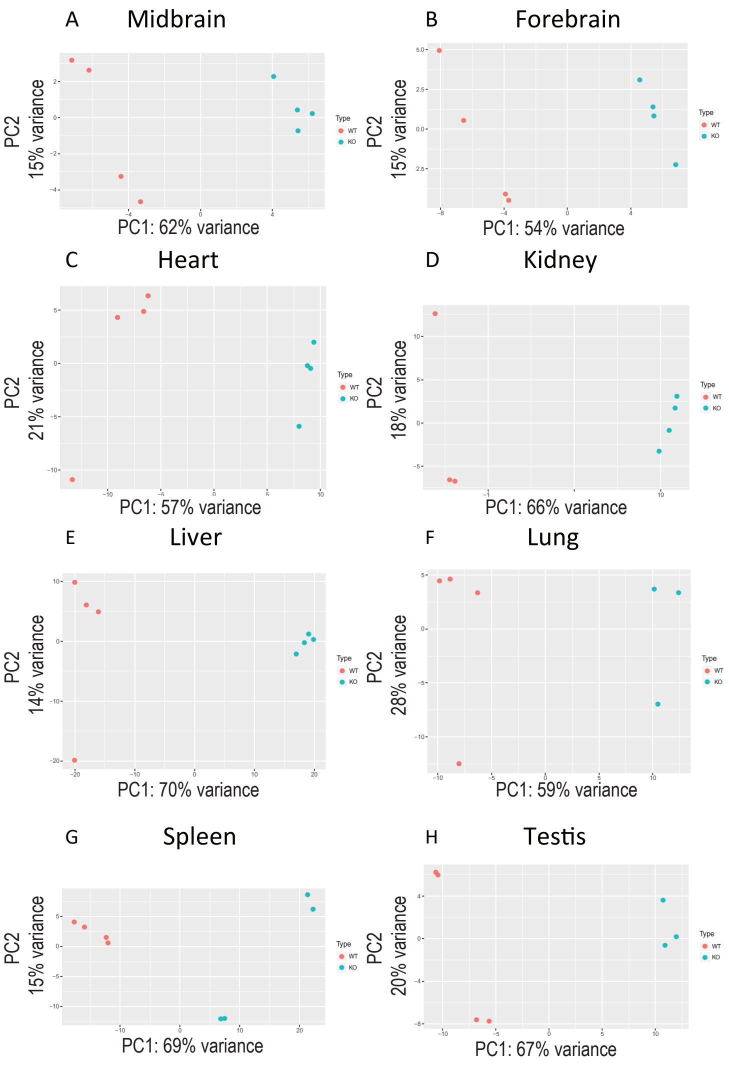
A
Midbrain
PC2
15% variance
PC1: 62% variance
Type
WT
KO
B
Forebrain
PC2
15% variance
PC1: 54% variance
Type
WT
KO
C
Heart
PC2
21% variance
PC1: 57% variance
Type
WT
KO
D
Kidney
PC2
18% variance
PC1: 66% variance
Type
WT
KO
E
Liver
PC2
14% variance
PC1: 70% variance
Type
WT
KO
F
Lung
PC2
28% variance
PC1: 59% variance
Type
WT
KO
G
Spleen
PC2
15% variance
PC1: 69% variance
Type
WT
KO
H
Testis
PC2
20% variance
PC1: 67% variance
Type
WT
KO

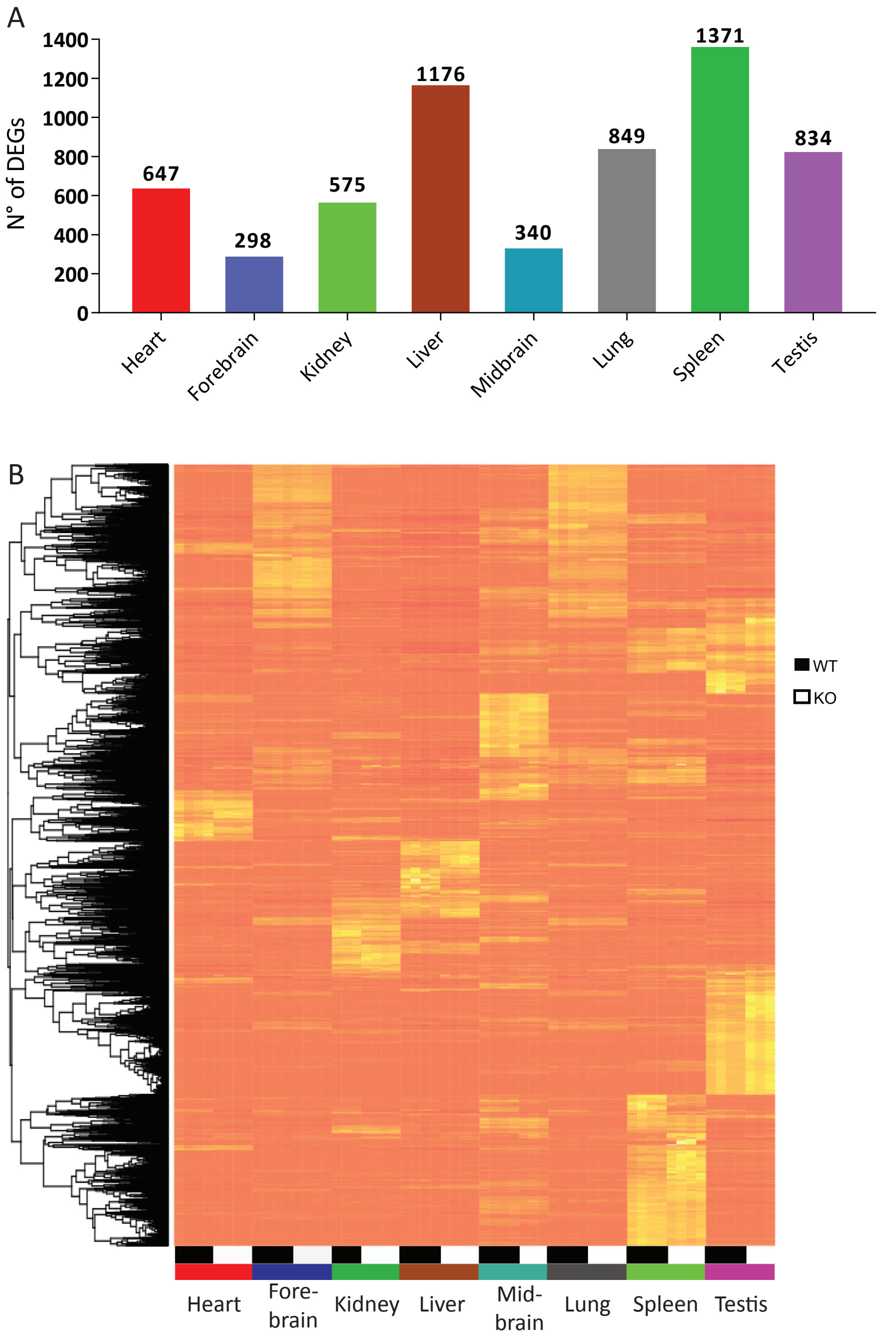

A
N° of DEGs
1400
1200
1000
800
600
400
200
0
647
298
575
1176
340
849
1371
834
Heart
Forebrain
Kidney
Liver
Midbrain
Lung
Spleen
Testis
B
WT
KO
Heart
Fore-brain
Kidney
Liver
Mid-brain
Lung
Spleen
Testis

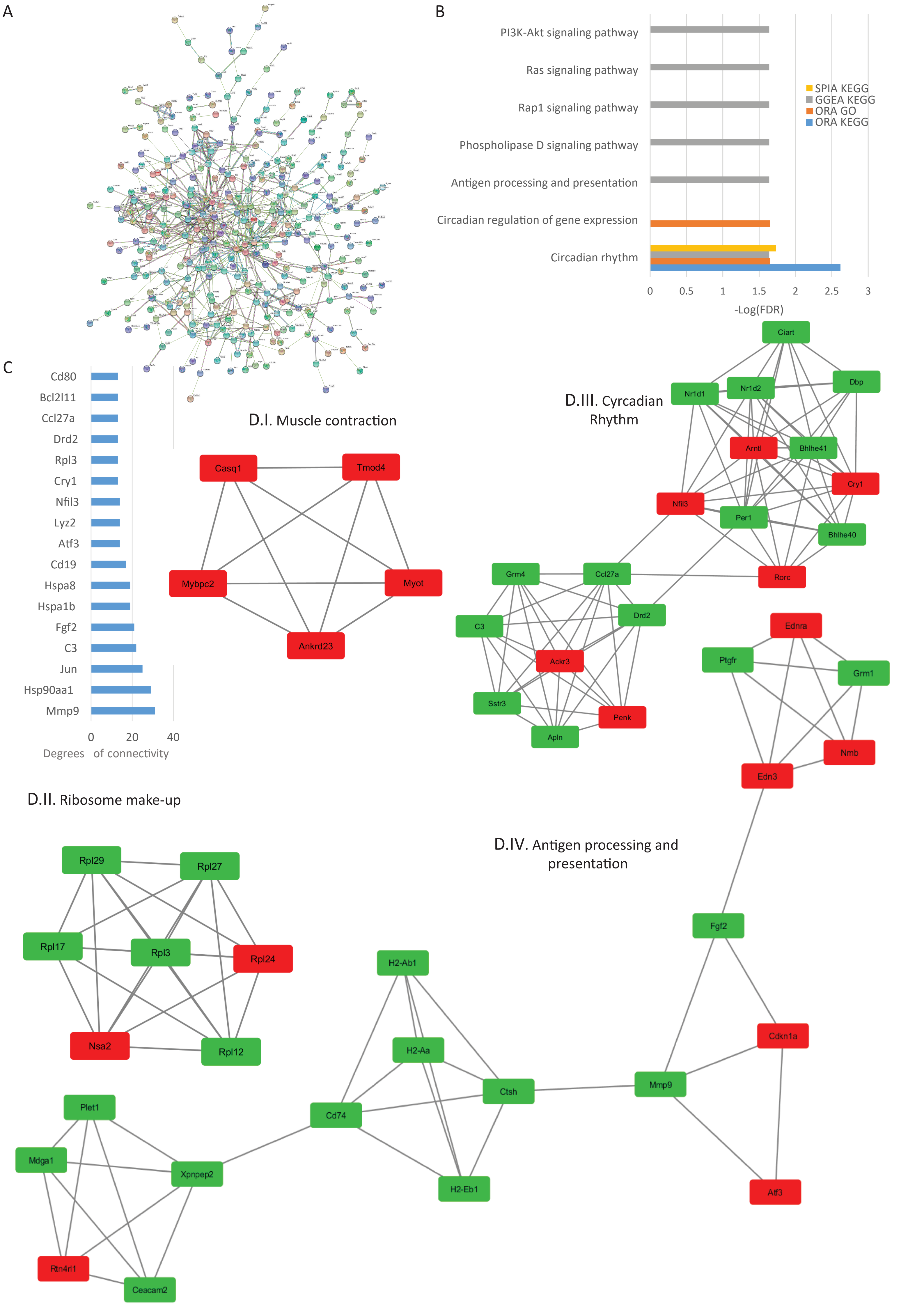
A
B
PI3K-Akt signaling pathway
Ras signaling pathway
Rap1 signaling pathway
Phospholipase D signaling pathway
Antigen processing and presentation
Circadian regulation of gene expression
Circadian rhythm
SPIA KEGG
GGEA KEGG
ORA GO
ORA KEGG
0
0.5
1
1.5
2
2.5
3
-Log(FDR)
C
Cd80
Bcl2l11
Ccl27a
Drd2
Rpl3
Cry1
Nfil3
Lyz2
Atf3
Cd19
Hspa8
Hspa1b
Fgf2
C3
Jun
Hsp90aa1
Mmp9
0
20
40
Degrees of connectivity
D.I. Muscle contraction
Casq1
Tmod4
Mybpc2
Myot
Ankrd23
D.III. Cyrcadian Rhythm
Ciart
Nr1d1
Nr1d2
Dbp
Arntl
Bhlhe41
Cry1
Nfil3
Per1
Bhlhe40
Rorc
Grm4
Ccl27a
Drd2
C3
Ackr3
Sstr3
Penk
Apln
Ednra
Ptgfr
Grm1
Nmb
Edn3
D.II. Ribosome make-up
Rpl29
Rpl27
Rpl17
Rpl3
Rpl24
Nsa2
Rpl12
D.IV. Antigen processing and presentation
Fgf2
H2-Ab1
H2-Aa
Cdkn1a
Ctsh
Mmp9
Cd74
Plet1
Mdga1
Xpnpep2
H2-Eb1
Atf3
Rtn4rl1
Ceacam2

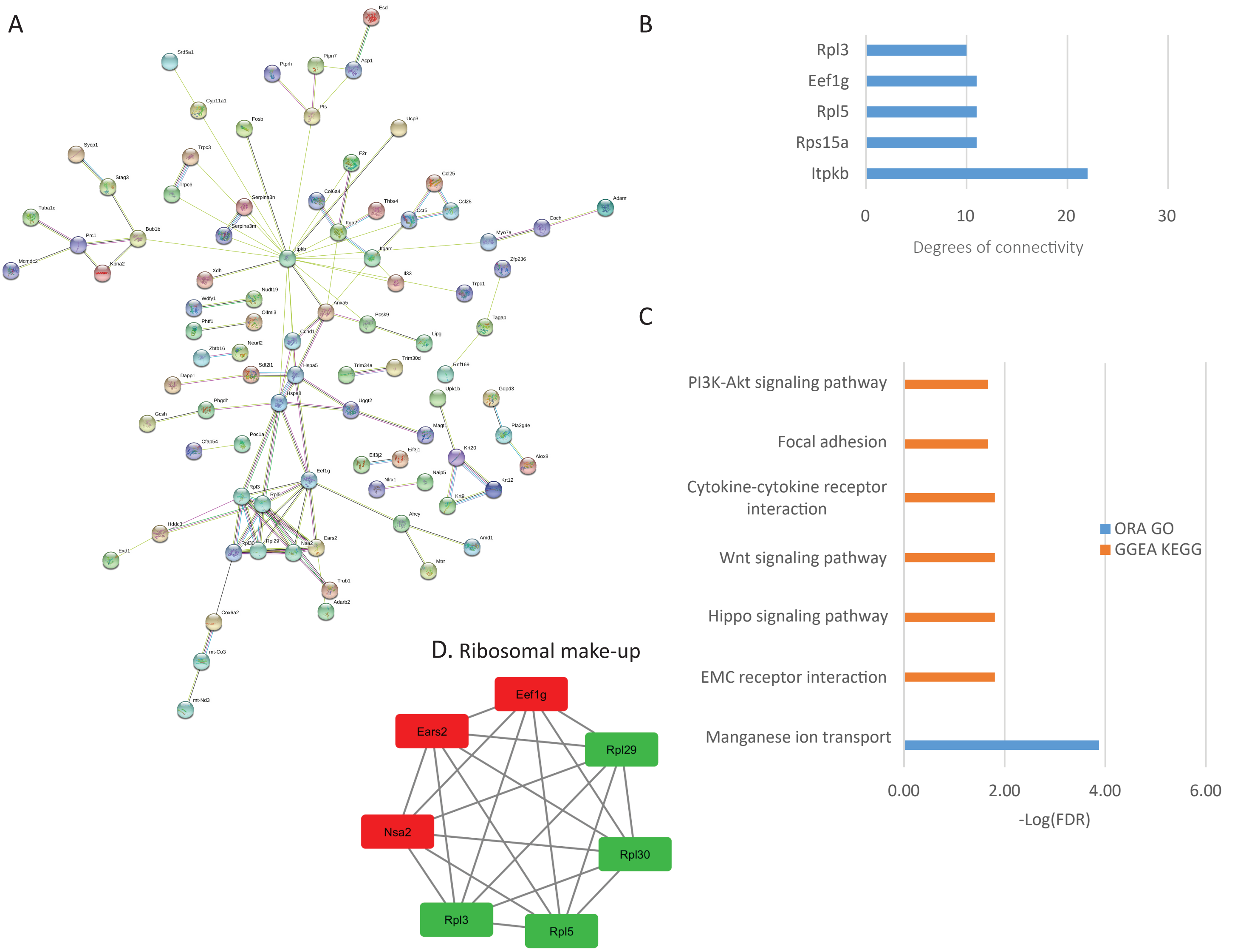
A
B
Rpl3
Eef1g
Rpl5
Rps15a
Itpkb
0
10
20
30
Degrees of connectivity
C
PI3K-Akt signaling pathway
Focal adhesion
Cytokine-cytokine receptor interaction
Wnt signaling pathway
Hippo signaling pathway
EMC receptor interaction
Manganese ion transport
ORA GO
GGEA KEGG
0.00
2.00
4.00
6.00
-Log(FDR)
D. Ribosomal make-up
Eef1g
Ears2
Rpl29
Nsa2
Rpl30
Rpl3
Rpl5

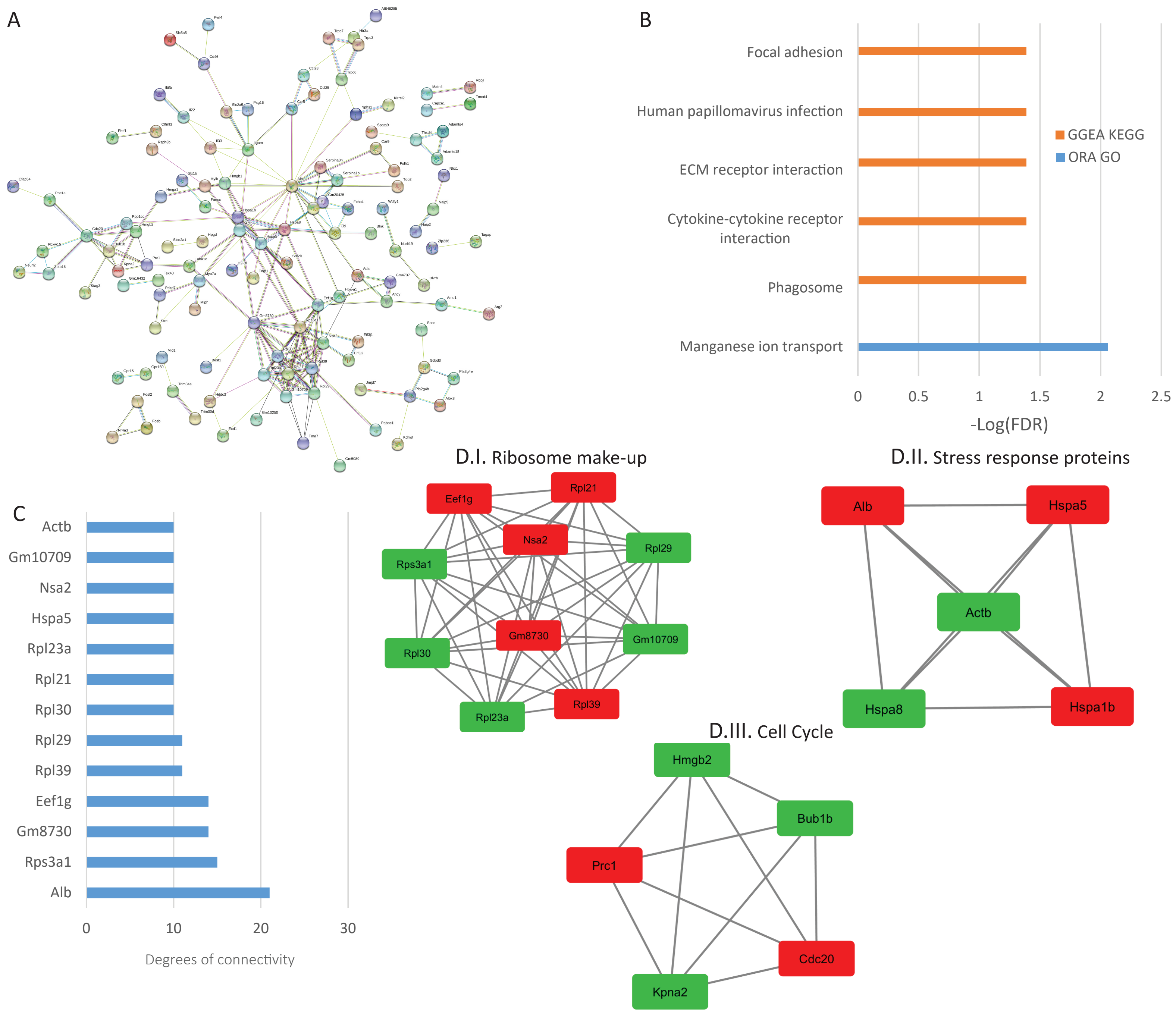

A
B
Focal adhesion
Human papillomavirus infection
ECM receptor interaction
Cytokine-cytokine receptor interaction
Phagosome
Manganese ion transport
GGEA KEGG
ORA GO
0
0.5
1
1.5
2
2.5
-Log(FDR)
C
Actb
Gm10709
Nsa2
Hspa5
Rpl23a
Rpl21
Rpl30
Rpl29
Rpl39
Eef1g
Gm8730
Rps3a1
Alb
0
10
20
30
Degrees of connectivity
D.I. Ribosome make-up
Eef1g
Rpl21
Nsa2
Rpl29
Rps3a1
Gm8730
Gm10709
Rpl30
Rpl39
Rpl23a
D.II. Stress response proteins
Alb
Hspa5
Actb
Hspa8
Hspa1b
D.III. Cell Cycle
Hmgb2
Bub1b
Prc1
Cdc20
Kpna2

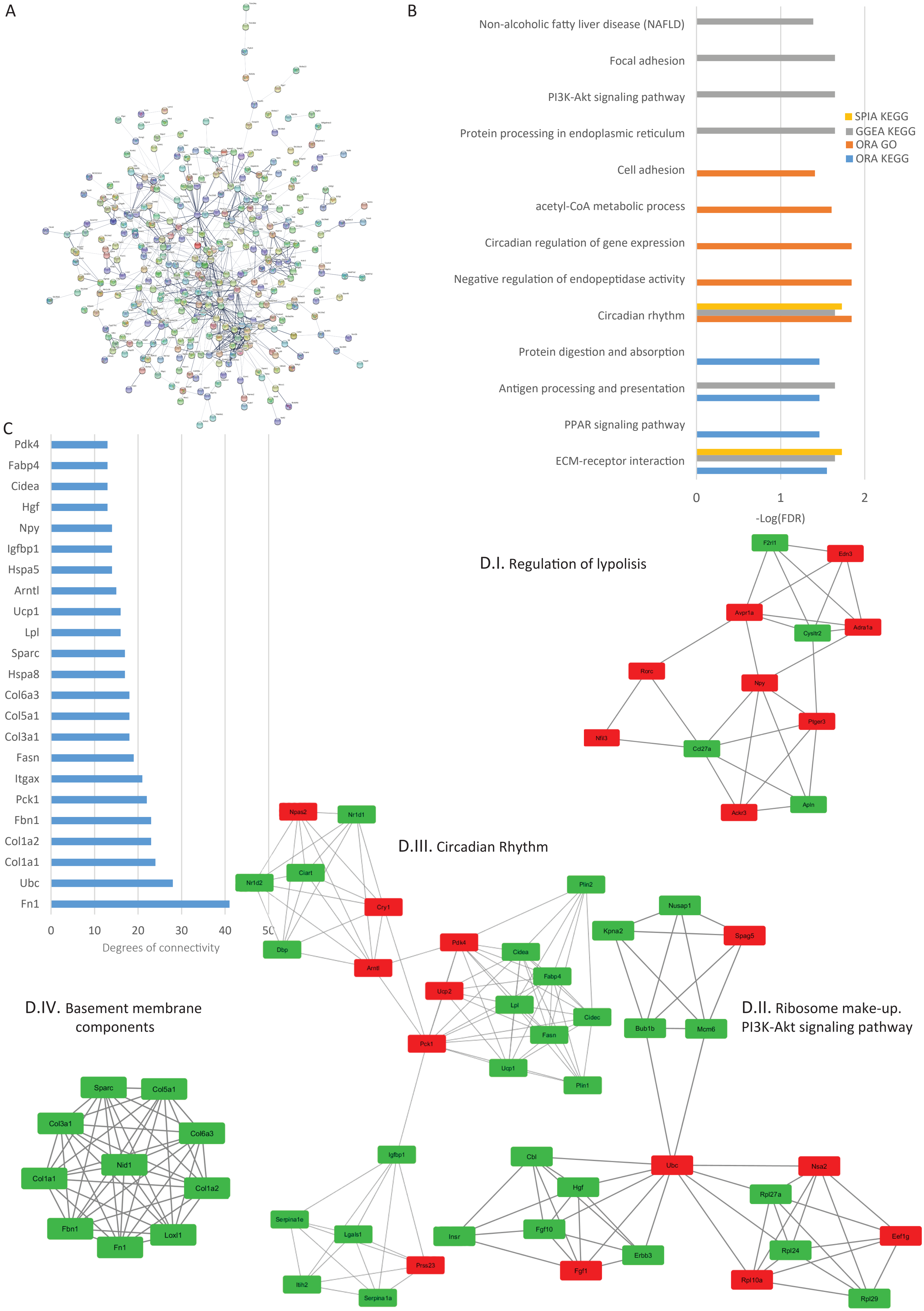
A
B
Non-alcoholic fatty liver disease (NAFLD)
Focal adhesion
PI3K-Akt signaling pathway
Protein processing in endoplasmic reticulum
Cell adhesion
acetyl-CoA metabolic process
Circadian regulation of gene expression
Negative regulation of endopeptidase activity
Circadian rhythm
Protein digestion and absorption
Antigen processing and presentation
PPAR signaling pathway
ECM-receptor interaction
SPIA KEGG
GGEA KEGG
ORA GO
ORA KEGG
0
1
2
-Log(FDR)
C
Pdk4
Fabp4
Cidea
Hgf
Npy
Igfbp1
Hspa5
Arntl
Ucp1
Lpl
Sparc
Hspa8
Col6a3
Col5a1
Col3a1
Fasn
Itgax
Pck1
Fbn1
Col1a2
Col1a1
Ubc
Fn1
0
10
20
30
40
50
Degrees of connectivity
D.I. Regulation of lypolisis
F2rl1
Edn3
Avpr1a
Cysltr2
Adra1a
Rorc
Npy
Ptger3
Nfil3
Cd27a
Ackr3
Apln
D.III. Circadian Rhythm
Npas2
Nr1d1
Ciart
Nr1d2
Cry1
Dbp
Arntl
Plin2
Pdk4
Cidea
Fabp4
Ucp2
Lpl
Cidec
Fasn
Pck1
Ucp1
Plin1
Nusap1
Kpna2
Spag5
Bub1b
Mcm6
D.II. Ribosome make-up.
PI3K-Akt signaling pathway
D.IV. Basement membrane
components
Sparc
Col5a1
Col3a1
Col6a3
Nid1
Col1a1
Col1a2
Fbn1
Fn1
Loxl1
Igfbp1
Serpina1e
Lgals1
Prss23
Itih2
Serpina1a
Cbl
Hgf
Ubc
Nsa2
Rpl27a
Insr
Fgf10
Erbb3
Fgf1
Eef1g
Rpl24
Rpl10a
Rpl29

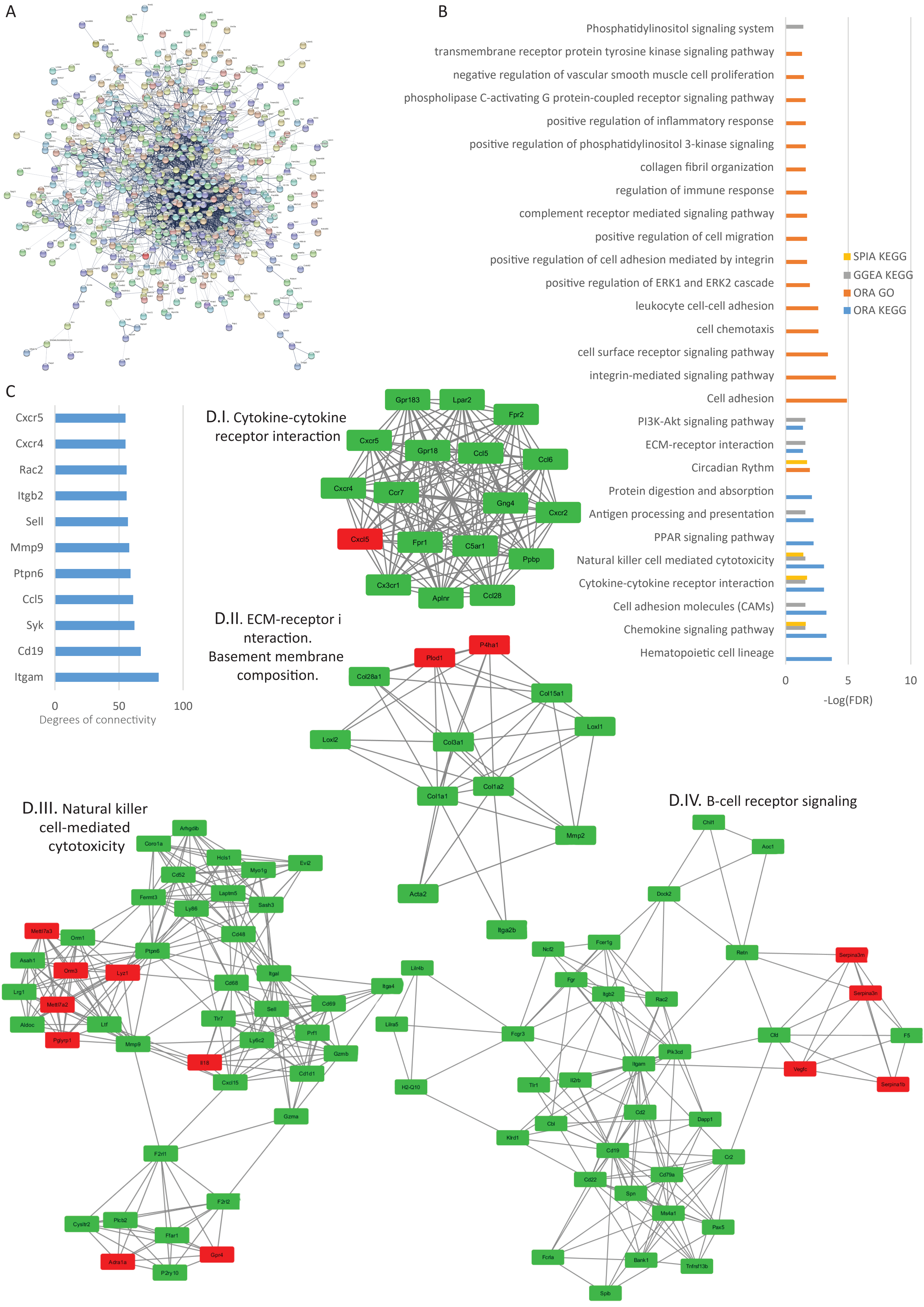
A
B
Phosphatidylinositol signaling system
transmembrane receptor protein tyrosine kinase signaling pathway
negative regulation of vascular smooth muscle cell proliferation
phospholipase C-activating G protein-coupled receptor signaling pathway
positive regulation of inflammatory response
positive regulation of phosphatidylinositol 3-kinase signaling
collagen fibril organization
regulation of immune response
complement receptor mediated signaling pathway
positive regulation of cell migration
positive regulation of cell adhesion mediated by integrin
positive regulation of ERK1 and ERK2 cascade
leukocyte cell-cell adhesion
cell chemotaxis
cell surface receptor signaling pathway
integrin-mediated signaling pathway
Cell adhesion
PI3K-Akt signaling pathway
ECM-receptor interaction
Circadian Rythm
Protein digestion and absorption
Antigen processing and presentation
PPAR signaling pathway
Natural killer cell mediated cytotoxicity
Cytokine-cytokine receptor interaction
Cell adhesion molecules (CAMs)
Chemokine signaling pathway
Hematopoietic cell lineage
SPIA KEGG
GGEA KEGG
ORA GO
ORA KEGG
0
5
10
-Log(FDR)
C
Cxcr5
Cxcr4
Rac2
Itgb2
Sell
Mmp9
Ptpn6
Ccl5
Syk
Cd19
Itgam
0
50
100
Degrees of connectivity
D.I. Cytokine-cytokine receptor interaction
Gpr183
Lpar2
Fpr2
Cxcr5
Gpr18
Ccl5
Ccl6
Cxcr4
Ccr7
Gng4
Cxcr2
Cxcl5
Fpr1
C5ar1
Ppbp
Cx3cr1
Aplnr
Ccl28
D.II. ECM-receptor i nteraction. Basement membrane composition.
P4ha1
Plod1
Col28a1
Col15a1
Loxl1
Loxl2
Col3a1
Col1a2
Col1a1
Mmp2
Acta2
Itga2b
D.III. Natural killer cell-mediated cytotoxicity
Arhgdib
Coro1a
Hcls1
Evi2
Myo1g
Cd52
Fermt3
Laptm5
Sash3
Ly86
Cd48
Mettl7a3
Orm1
Asah1
Orm3
Lyz1
Ptpn6
Itgal
Lrg1
Mettl7a2
Cd68
Itga4
Cd69
Tlr7
Sell
Aldoc
Ltf
Prf1
Pglyrp1
Mmp9
Ly6c2
Gzmb
Il18
Cd1d1
Cxcl15
Gzma
F2rl1
F2rl2
Cysltr2
Plcb2
Ffar1
Adra1a
Gpr4
P2ry10
D.IV. B-cell receptor signaling
Chil1
Aoc1
Dock2
Ncf2
Fcer1g
Retn
Serpina3m
Lilr4b
Fgr
Itgb2
Rac2
Serpina3n
Lilra5
Fcgr3
Cfd
F5
Pik3cd
Itgam
Vegfc
Tlr1
Il2rb
Serpina1b
H2-Q10
Cd2
Dapp1
Cbl
Klrd1
Cd19
Cr2
Cd79a
Cd22
Spn
Ms4a1
Pax5
Fcrla
Bank1
Tnfrsf13b
Spib

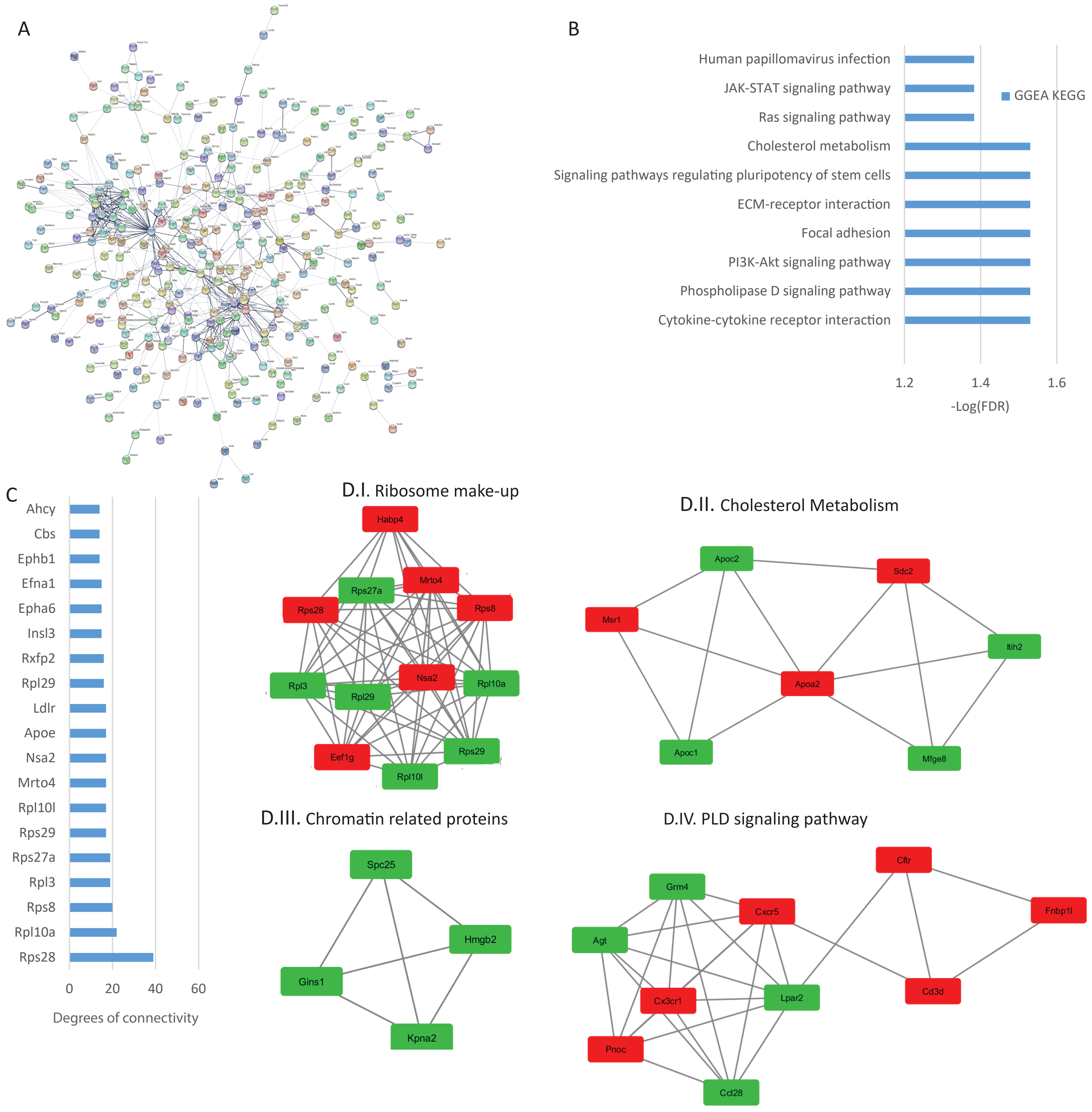
A
B
Human papillomavirus infection
JAK-STAT signaling pathway
Ras signaling pathway
Cholesterol metabolism
Signaling pathways regulating pluripotency of stem cells
ECM-receptor interaction
Focal adhesion
PI3K-Akt signaling pathway
Phospholipase D signaling pathway
Cytokine-cytokine receptor interaction
GGEA KEGG
1.2
1.4
1.6
-Log(FDR)
C
Ahcy
Cbs
Ephb1
Efna1
Epha6
Insl3
Rxfp2
Rpl29
Ldlr
Apoe
Nsa2
Mrto4
Rpl10l
Rps29
Rps27a
Rpl3
Rps8
Rpl10a
Rps28
0
20
40
60
Degrees of connectivity
D.I. Ribosome make-up
Habp4
Rps27a
Mrto4
Rps28
Rps8
Rpl3
Rpl29
Nsa2
Rpl10a
Eef1g
Rpl10l
Rps29
D.II. Cholesterol Metabolism
Apoc2
Sdc2
Msr1
Itih2
Apoa2
Apoc1
Mfge8
D.III. Chromatin related proteins
Spc25
Hmgb2
Gins1
Kpna2
D.IV. PLD signaling pathway
Cftr
Grm4
Cxcr5
Fnbp1l
Agt
Cx3cr1
Lpar2
Cd3d
Pnoc
Ccl28

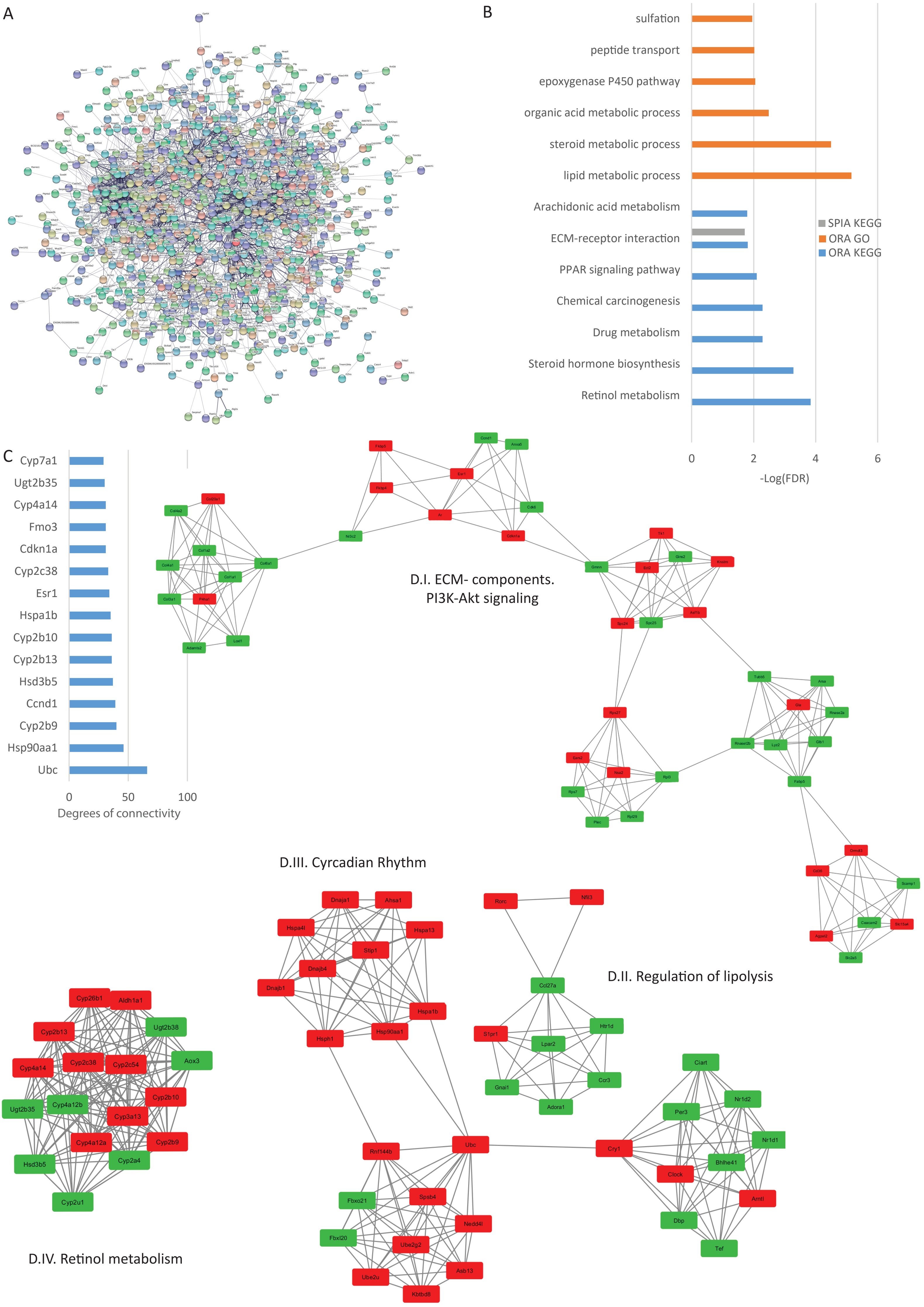

A
B
sulfation
peptide transport
epoxygenase P450 pathway
organic acid metabolic process
steroid metabolic process
lipid metabolic process
Arachidonic acid metabolism
ECM-receptor interaction
PPAR signaling pathway
Chemical carcinogenesis
Drug metabolism
Steroid hormone biosynthesis
Retinol metabolism
SPIA KEGG
ORA GO
ORA KEGG
0
2
4
6
-Log(FDR)
C
Cyp7a1
Ugt2b35
Cyp4a14
Fmo3
Cdkn1a
Cyp2c38
Esr1
Hspa1b
Cyp2b10
Cyp2b13
Hsd3b5
Ccnd1
Cyp2b9
Hsp90aa1
Ubc
0
50
100
Degrees of connectivity
D.I. ECM- components.
PI3K-Akt signaling
D.II. Regulation of lipolysis
D.III. Cyrcadian Rhythm
D.IV. Retinol metabolism
Dnaja1
Ahsa1
Hspa4l
Hspa13
Stip1
Dnajb4
Dnajb1
Hspa1b
Hsph1
Hsp90aa1
Rorc
Nfil3
Ccl27a
S1pr1
Htr1d
Lpar2
Gnai1
Ccr3
Adora1
Cyp26b1
Aldh1a1
Cyp2b13
Ugt2b38
Aox3
Cyp4a14
Cyp2c38
Cyp2c54
Cyp2b10
Ugt2b35
Cyp4a12b
Cyp3a13
Cyp4a12a
Cyp2b9
Hsd3b5
Cyp2a4
Cyp2u1
Rnf144b
Ubc
Cry1
Ciart
Nr1d2
Per3
Nr1d1
Bhlhe41
Clock
Arntl
Dbp
Tef
Fbxo21
Spsb4
Nedd4l
Fbxl20
Ube2g2
Asb13
Ube2u
Kbtbd8

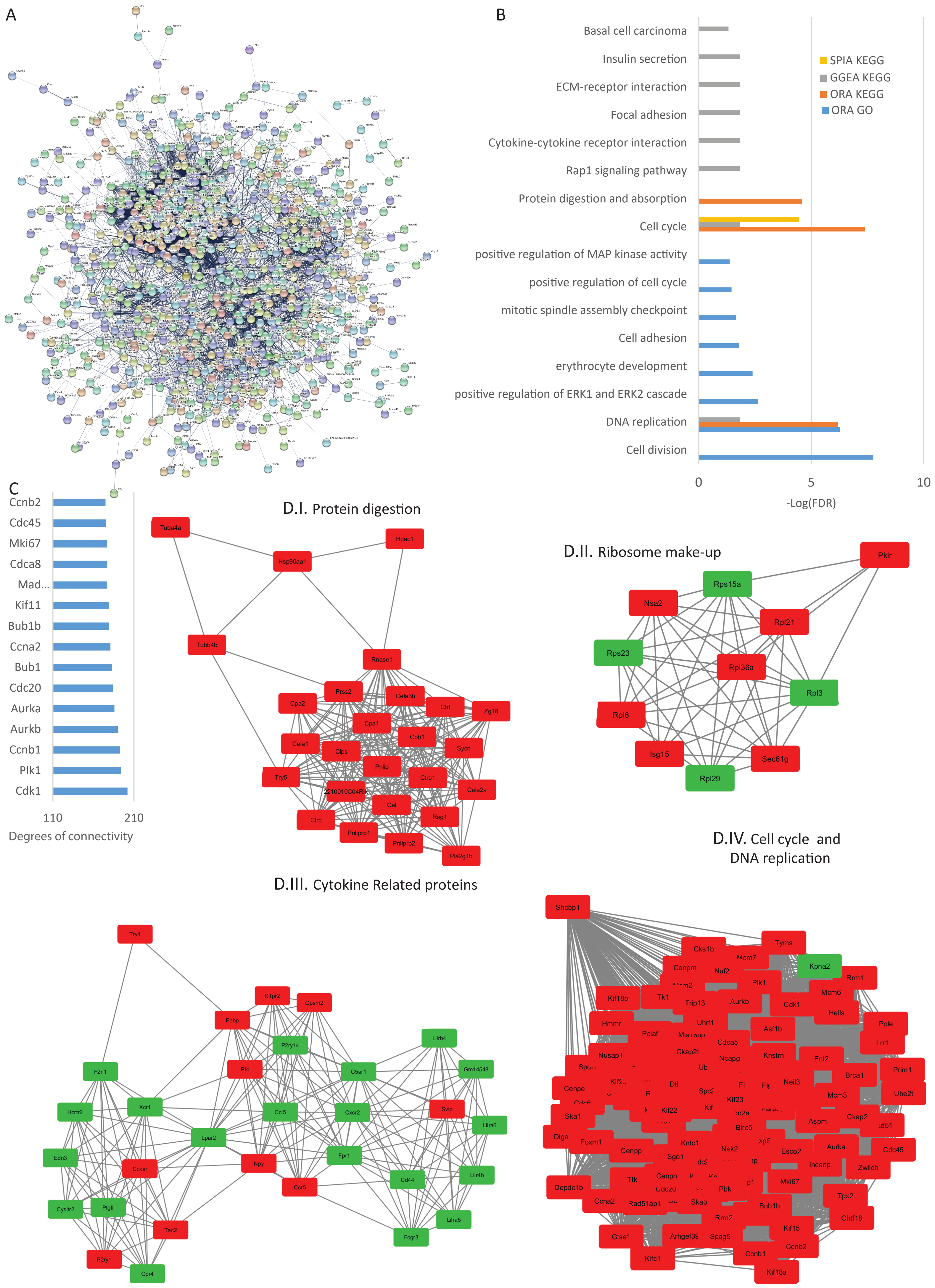

A
B
Basal cell carcinoma
Insulin secretion
ECM-receptor interaction
Focal adhesion
Cytokine-cytokine receptor interaction
Rap1 signaling pathway
Protein digestion and absorption
Cell cycle
positive regulation of MAP kinase activity
positive regulation of cell cycle
mitotic spindle assembly checkpoint
Cell adhesion
erythrocyte development
positive regulation of ERK1 and ERK2 cascade
DNA replication
Cell division
SPIA KEGG
GGEA KEGG
ORA KEGG
ORA GO
0
5
10
-Log(FDR)
C
Ccnb2
Cdc45
Mki67
Cdca8
Mad...
Kif11
Bub1b
Ccna2
Bub1
Cdc20
Aurka
Aurkb
Ccnb1
Plk1
Cdk1
110
210
Degrees of connectivity
D.I. Protein digestion
Tuba4a
Hdac1
Hsp90aa1
Tubb4b
Rnase1
Prss2
Cpa2
Cela3b
Ctrl
Zg16
Cpa1
Cpb1
Cela1
Clps
Sycn
Pnlip
Try5
Ctrb1
Cela2a
2210010C04Rik
Cel
Ctrc
Reg1
Pnliprp1
Pnliprp2
Pla2g1b
D.II. Ribosome make-up
Pklr
Rps15a
Nsa2
Rpl21
Rps23
Rpl36a
Rpl3
Rpl6
Isg15
Sec61g
Rpl29
D.III. Cytokine Related proteins
Try4
S1pr2
Gpsm2
Ppbp
P2ry14
Lilrb4
F2rl1
Pf4
C5ar1
Gm14548
Hcrtr2
Xcr1
Ccl5
Cxcr2
Svip
Lilra6
Lpar2
Edn3
Cckar
Npy
Fpr1
Cd44
Lilr4b
Ccr5
Cysltr2
Ptgfr
Tac2
Lilra5
Fcgr3
P2ry1
Gpr4
D.IV. Cell cycle and DNA replication
Shcbp1
Tyms
Cks1b
Mcm7
Kpna2
Cenpm
Nuf2
Rrm1
Plk1
Kif18b
Tk1
Trip13
Aurkb
Cdk1
Mcm6
Hells
Hmmr
Uhrf1
Pole
Pclaf
Asf1b
Lrr1
Cdca5
Nusap1
Ckap2l
Knstn
Ect2
Ncapg
Prim1
Brca1
Neil3
Cenpe
Dtl
Mcm3
Ube2t
Kif23
Ska1
Kif22
Ckap2
Aspm
Birc5
Dlga
Foxm1
Kntc1
Cenpp
Nek2
Esco2
Aurka
Cdc45
Sgo1
Incenp
Zwilch
Depdc1b
Ttk
Cenpn
Mki67
Pbk
Tpx2
Ccna2
Rad51ap1
Ska3
Bub1b
Rrm2
Chtf18
Kif15
Gtse1
Arhgef39
Spag5
Ccnb2
Ccnb1
Kifc1
Kif18a

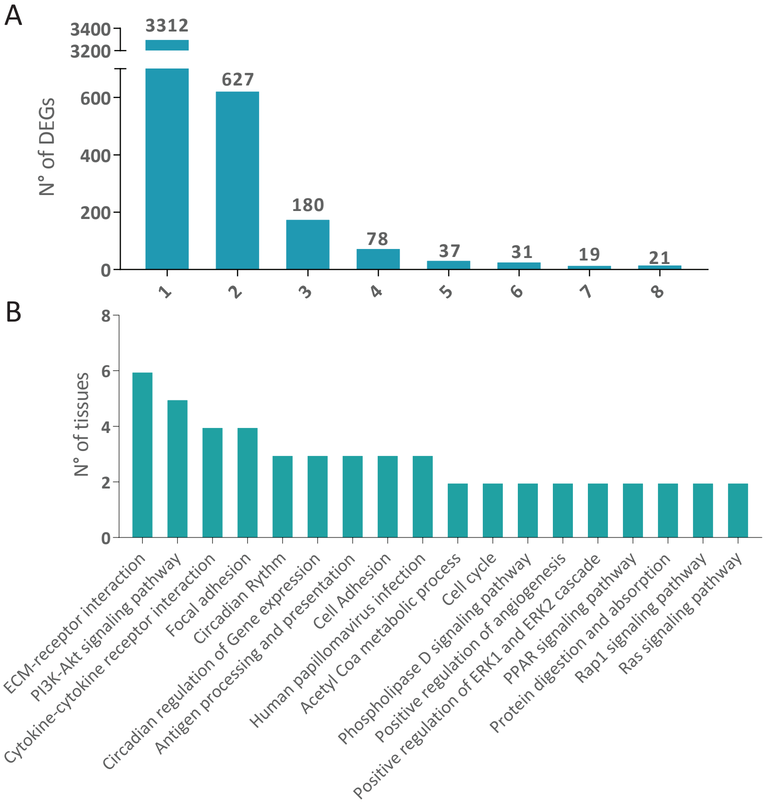
A
N° of DEGs
3400
3200
600
400
200
0
3312
627
180
78
37
31
19
21
1
2
3
4
5
6
7
8
B
N° of tissues
8
6
4
2
0
ECM-receptor interaction
PI3K-Akt signaling pathway
Cytokine-cytokine receptor interaction
Focal adhesion
Circadian Rythm
Circadian regulation of Gene expression
Antigen processing and presentation
Cell Adhesion
Human papillomavirus infection
Acetyl Coa metabolic process
Cell cycle
Phospholipase D signaling pathway
Positive regulation of angiogenesis
Positive regulation of ERK1 and ERK2 cascade
PPAR signaling pathway
Protein digestion and absorption
Rap1 signaling pathway
Ras signaling pathway
